# Large-scale analyses identify a cluster of novel long noncoding RNAs as potential competitive endogenous RNAs in progression of hepatocellular carcinoma

**DOI:** 10.18632/aging.102468

**Published:** 2019-11-23

**Authors:** Mengjia Song, Ailin Zhong, Jieying Yang, Junyi He, Shaoyan Cheng, Jianxiong Zeng, Yue Huang, Qiuzhong Pan, Jingjing Zhao, Ziqi Zhou, Qian Zhu, Yan Tang, Hao Chen, Chaopin Yang, Yuan Liu, Xiaocong Mo, Desheng Weng, Jian-Chuan Xia

**Affiliations:** 1 Department of Biotherapy, Sun Yat-sen University Cancer Center, Guangzhou, P. R. China; 2 Collaborative Innovation Center for Cancer Medicine, State Key Laboratory of Oncology in South China, Sun Yat-sen University Cancer Center, Guangzhou, China; 3 Biotherapy Center, The First Affiliated Hospital of Zhengzhou University, Zhengzhou, China; 4 Office of International Exchange and Cooperation, Guangzhou University of Chinese Medicine, Guangzhou, People's Republic of China

**Keywords:** CeRNA, lncRNA, hepatocellular carcinoma, progression

## Abstract

The abnormal expression of noncoding RNAs has attracted increasing interest in the field of hepatocellular carcinoma progression. However, the underlying molecular mechanisms mediated by noncoding RNAs in these processes are unclear. Here, we obtained the expression profiles of long noncoding RNAs, microRNAs, and mRNAs from the Gene Expression Omnibus database and identified hepatocarcinogenesis-specific differentially expressed transcripts. Next, we identified significant Gene Ontology and pathway terms that the differentially expressed transcripts involved in. Using functional analysis and target prediction, we constructed a hepatocellular carcinoma-associated deregulated competitive endogenous RNA network to reveal the potential mechanisms underlying tumor progression. By analyzing The Cancer Genome Atlas dataset, six key long noncoding RNAs showed significant association with overall survival as well as strong correlation with some microRNAs and mRNAs in the competitive endogenous RNA network. We further validated the above results and determined their diagnostic and prognostic value in clinical samples. Importantly, by large-scale analyses, we identified a cluster of long noncoding RNAs, *GBAP1*, *MCM3AP-AS1*, *SLC16A1-AS1*, *C3P1*, *DIO3OS*, and *HNF4A-AS1* as candidate biomarkers for the diagnosis and prognosis of hepatocellular carcinoma, which will improve our understanding of competitive endogenous RNA-mediated regulatory mechanisms underlying hepatocellular carcinoma development and will provide novel therapeutic targets in the future.

## INTRODUCTION

Hepatocellular carcinoma (HCC) is one of the fatal malignant tumors worldwide, especially in Asia [1]. Despite advances in prevention, detection, diagnosis, and treatment of HCC in recent years, its incidence and mortality are increasing significantly every year, and the five-year overall survival (OS) rate is only 18% [2]. The development of HCC is a complex multistep process involving a series of molecular pathogeneses and multiple factors; therefore, there is an urgent need to identify more detailed mechanisms to improve the outcome of patients with HCC [3]. Recent genomic researches have identified many oncogenes as indispensable factors involved in the development of various types of cancer, including HCC, which provide more advanced diagnostic approaches and new targets for cancer treatment [4, 5].

Long non-coding RNAs (lncRNAs) comprise transcripts with a length of over 200 nucleotides, which are characterized by low expression in cancer, high expression in tissues, and cell-specificity [6]. Despite their inability to encode proteins, lncRNAs regulate the expression of many mRNAs by acting in cis and in trans. Cis-acting lncRNAs influence the expression and chromatin state of nearby genes via altering their transcription, recruiting regulatory factors, and splicing of the lncRNA. They also rely on DNA elements within the lncRNA promoter or gene locus. However, trans-acting lncRNAs leave the site of transcription and execute an array of functions throughout the cell by influencing nuclear structure and organization, as well as regulating the behavior of proteins and other RNA molecules [7]. As a result, the dysregulation of lncRNA expression affects cellular homeostasis, which might lead to cancer initiation and progression [6, 8]. MicroRNAs (miRNAs) are a type of small non-coding RNAs composed of 21–22 nucleotides. They exert their biological effects by silencing genes post-transcriptionally via binding to miRNA response elements (MREs) in the target mRNA [9]. Recently, the theory of a competing endogenous RNA (ceRNA) regulation network in cancer has been proposed [10]. This hypothesis states that ceRNAs harbor MREs and bind to miRNAs in competition with their target mRNAs, leading to blockade of the silencing effect of miRNAs on their target mRNAs. Accumulating studies have confirmed that lncRNAs act as sponges to sequester and bind miRNAs in competition with mRNAs [11]. Therefore, lncRNA can be considered as a kind of ceRNA that regulates transcript expression. This theory has been proposed in different types of cancer. For example, Liang et al*.* constructed a lncRNA-mediated ceRNA network for mesenchymal ovarian cancer and showed that lncRNA *PTAR* acted as a ceRNA to promote epithelial-mesenchymal transition (EMT), invasion, and metastasis by competitively binding to miR-101-3p to regulate *ZEB1* expression [12]. Another study identified transforming growth factor-beta (TGF-β) promoted tumor invasion and metastasis by downregulating *EPB41L4A-AS2*, a novel lncRNA functioning as a ceRNA in head and neck squamous cell carcinoma [13]. In HCC, Sui et al*.* reported that lncRNA *GIHCG* promoted HCC cell proliferation, migration, and invasion *in vitro* and xenograft growth and metastasis *in vivo* depending on its silencing of miR-200b/a/429 [14]. Ren et al*.* proposed the TP53-miR-215-PCAT-1-CRKL axis as an important regulatory pathway inhibiting tumor cell proliferation, migration, and invasion in HCC [15]. However, whether the ceRNA network mediates HCC initiation and progression remains unclear.

The present study aimed to reveal the potential regulatory mechanisms involved in HCC development by constructing an mRNA-miRNA-lncRNA interaction network. We comprehensively analyzed the regulatory network of HCC-related genes, miRNAs, and lncRNAs based on the gene expression profile, including differential expression profiles analysis, gene ontology (GO) enrichment, Kyoto Encyclopedia of Genes and Genomes (KEGG) pathway analysis, and signal regulation network (signal-net) construction. Based on these results and target transcripts prediction, we built a ceRNA network to indicate the key mRNA-miRNA-lncRNA interactions. Furthermore, a protein regulation network was used to clarify the regulatory interactions among these transcripts in the ceRNA network. For validation, we performed Pearson correlation analysis and survival analysis for the mRNAs, miRNAs, and lncRNAs identified in the ceRNA network in HCC tissues from The Cancer Genome Atlas (TCGA) database and clinical samples, respectively. The identified ceRNA network might provide potential biomarkers for predicting the prognosis and novel therapeutic targets for the treatment of patients with HCC.

## RESULTS

### Differential and clustering analysis

**Figure 1:**
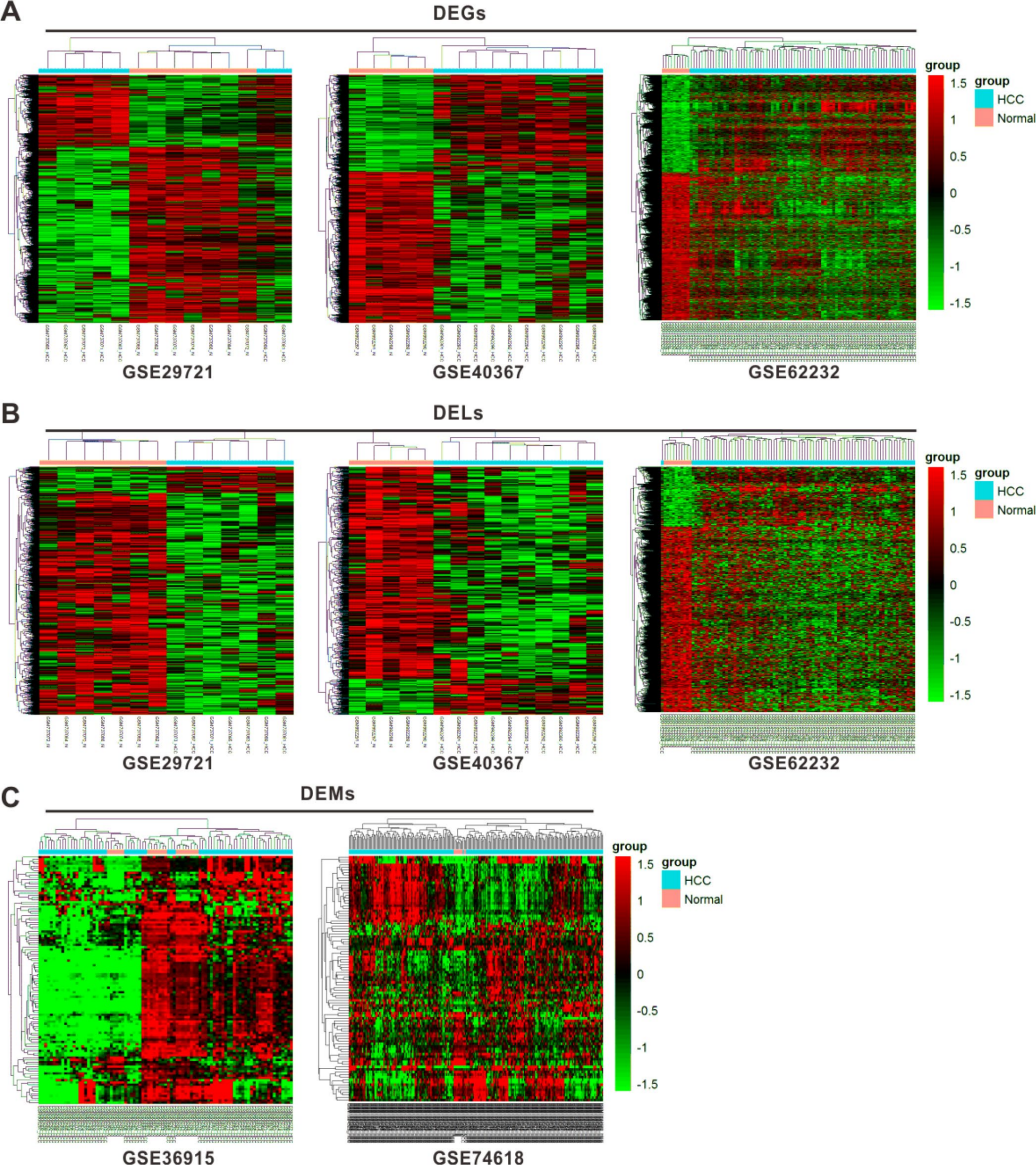
**Cluster analysis of differentially expressed profiles.** Hierarchical cluster dendrogram of DEGs (**A**) and DELs (**B**) identified in GES29721, GSE40367 and GSE62232 as well as DEMs (**C**) identified in GSE36915 and GSE74618. The rows showed DEGs, DELs, and DEMs, while the columns showed paired samples. The pink part represents normal samples and the blue part represents HCC samples. The left vertical axis shows clusters of DEGs, DELs and DEMs, while the above horizontal axis shows clusters of samples. Red represents high expression and green represents low expression.

The workflow chart was shown in Supplementary Figure 1. We first identified 11036 differentially expressed genes (DEGs) from three profiles, GSE29721, GSE40367, and GSE62232, consisting of 3949 (35.8%) upregulated and 7087 (64.2%) downregulated genes. The top 5 DEGs in the HCC samples compared with normal samples from different profiles were shown in Table 1 according to their fold change (FC) values. Besides, 3826 lncRNAs were selected as differentially expressed lncRNAs (DELs) from the same three profiles, including 615 (16.1%) upregulated and 3211 (83.9%) downregulated lncRNAs. From another two profiles, GSE36915 and GSE74618, 206 differentially expressed miRNAs (DEMs) were selected, including 80 (38.8%) upregulated and 126 (61.2%) downregulated miRNAs. The top 10 DELs and DEMs in the HCC samples in contrast to normal samples from different profiles were shown in Supplementary Tables 1 and 2, respectively. The results of hierarchical cluster analyses were shown in heatmaps for the expression level changes of DEGs (Figure 1A), DELs (Figure 1B), and DEMs (Figure 1C) between HCC and normal tissues.

**Table 1:** The top 5 upregulated and downregulated DEGs in different expression profiles.

**Profile**	**Gene symbol**	**Gene ID**	**style**	**Fold change**	***P*-value**
GSE29721	CCL20	6364	up	30.93373	0.000163
	HJURP	55355	up	23.79123	0.00002
	SPINK1	6690	up	22.69303	0.000635
	SULT1C2	6819	up	20.0141	0.0001927
	REG3A	5068	up	18.2934	0.005302
	MT1M	4499	down	-30.4032	0.001046
	TTC36	143941	down	-28.8462	0.000253
	THRSP	7069	down	-26.6209	0.0003503
	HAMP	57817	down	-26.0083	0.001316
	CLEC4M	10332	down	-22.2482	0.000288
GSE29721	CCL20	6364	up	106.6007	0.000019
	SPINK1	6690	up	47.69886	0.000022
	GABBR1	2550	up	43.14953	0.000018
	KIF20A	10112	up	36.35204	0.000019
	ELOVL7	79993	up	34.84073	0.00002
	CXCL14	9547	down	-132.845	0.0000185
	CYP1A2	1544	down	-101.348	0.000018
	CNDP1	84735	down	-87.8993	0.000018
	MT1M	4499	down	-62.8525	0.00002
	MME	4311	down	-60.0369	0.0000265
GSE62232	RPS4Y1	6192	up	90.17847	<0.00001
	AKR1B10	57016	up	34.80468	<0.00001
	CCL20	6364	up	26.06746	0.000001
	TOP2A	7153	up	20.6209	9.933E-05
	SPINK1	6690	up	18.16443	0.001901
	MT1M	4499	down	-75.7478	<0.00001
	CNDP1	84735	down	-70.9896	<0.00001
	CLEC4G	339390	down	-69.8899	<0.00001
	CXCL14	9547	down	-67.7081	3.333E-06
	CLEC4M	10332	down	-67.278	<0.00001

### Intersection analysis

Next, we selected 1016 intersecting DEGs and 116 intersecting DELs among the three profiles to perform further analysis. Twenty-one DEMs were also selected between the two profiles GSE36915 and GSE74618. The intersections were displayed as Venn diagrams (Supplementary Figure 2A–2C).

### GO and pathway analysis

**Figure 2:**
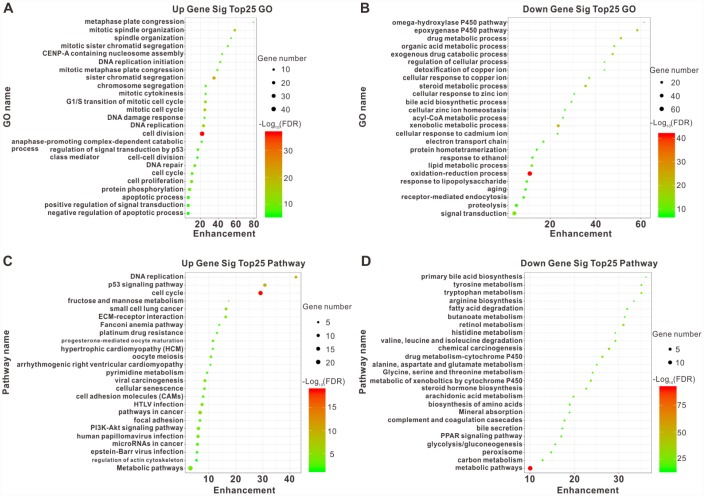
**Top25 enrichment of GO and KEGG pathway analyses for upregulated and downregulated DEGs.** The upregulated (**A**) and downregulated (**B**) DEGs enriched in GO categories. The upregulated (**C**) and downregulated (**D**) DEGs enriched in different pathways. The horizontal axis represents the enrichment score of DEGs. The vertical axis represents different GO categories and pathways. The bubble size indicates the number of genes in each category and pathway, and different colors correspond to different log (FDR) values.

According to the intersecting DEGs, we identified 1803 upregulated DEGs-related GO terms and 6339 downregulated DEGs-related GO terms using functional enrichment analysis. The plots of the top 25 upregulated and downregulated GO enrichment terms were shown in Figure 2A and 2B. The results demonstrated that the upregulated DEGs mainly participated in cell division, sister chromatid cohesion, mitotic spindle organization, DNA replication, and mitotic cell cycle; while the downregulated DEGs were closely associated with the oxidation-reduction process, xenobiotic metabolic process, and the epoxygenase P450 pathway.

We subsequently distinguished 221 pathways among the upregulated DEGs and 274 pathways among the downregulated DEGs using KEGG pathway analysis based on the intersecting DEGs. The plots of the top 25 pathways for the upregulated and downregulated DEGs were shown in Figure 2C and 2D. The most obviously upregulated pathways were the cell cycle, the p53 signaling pathway, and DNA replication. Pathways dramatically enriched in downregulated genes were metabolic pathways, chemical carcinogenesis, and retinol metabolism. Taken together, these dramatically changed GO terms and pathways might be involved in HCC initiation and progression.

### Signal-net analysis

To clarify the interactions among different genes and their products, we further constructed a gene signal-net using 1016 intersecting DEGs based on KEGG database according to the network biology theory. Signal-net could display the relationships between different gene groups and identify upstream and downstream molecules by obtaining gene interactions in multiple pathways. The network we constructed included 97 interacting DEGs, consisting of 23 upregulated genes and 74 downregulated genes (Supplementary Figure 3). According to their regulation degree, several DEGs were considered as the hub genes that exerted the most significant regulatory function in the network. For example, *PRKAA2*, which held the highest degree, had the strongest interactions with other genes in the network. The pathway information from the KEGG database indicated that *PRKAA2* and its related genes were involved in the AMPK/PI3K/AKT signaling pathways, a representative signaling pathway involved in the development of HCC [16]. Besides, several closely connected hub genes, including *ITGA6*, *ITGA2*, and *ITGB3*, are the main cellular adhesion receptors belonging to the integrins family and extensively implicated in multiple steps from cancer initiation to metastasis by acting as signaling molecules, mechanotransducers, and critical components of the cell migration machinery [17]. Therefore, DEGs with a high regulation degree in this signal-net, including *PRKAA2*, *PLCB1*, and several genes encoding the integrins family members might play crucial roles in the regulation of HCC development.

### CeRNA network

The above results demonstrated that some intersecting DEGs might play a crucial role in HCC development by participating in important processes, such as cell cycle, p53 signaling pathway, and metabolic pathways. We next investigated the underlying molecular mechanisms regulating these processes. LncRNAs have been reported to promote tumor progression through various types of gene regulatory mechanisms, such as epigenetic and transcriptional regulation and serving as ceRNAs for miRNAs [10, 11, 14, 15, 18–20]. Therefore, we constructed a ceRNA regulatory network to reveal the unknown mechanisms driving HCC development.

**Figure 3:**
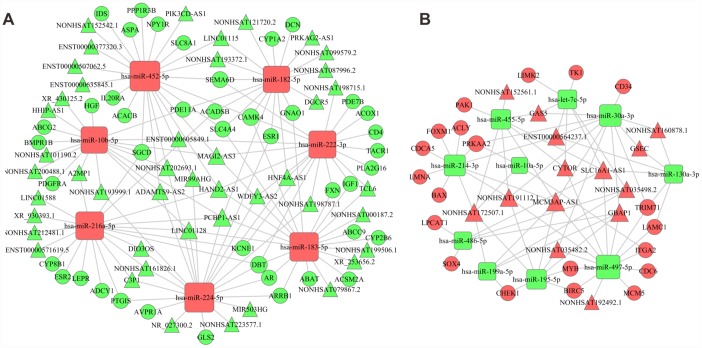
**The ceRNA network.** (**A**) Network constructed by upregulated miRNAs, downregulated lncRNAs and downregulated mRNAs. (**B**) Network constructed by downregulated miRNAs, upregulated lncRNAs and upregulated mRNAs. Red diamonds represent upregulated miRNAs, red balls, upregulated mRNAs, and red triangles, upregulated lncRNAs. Green diamonds represent downregulated miRNAs, green balls, downregulated mRNAs, green triangles, downregulated lncRNAs.

Intersection datasets were acquired between the DEGs involved in the significant enriched GO terms and KEGG pathways with *P* < 0.05 and false discovery rate (FDR) < 0.05, which included 393 DEGs. We predicted 213 target genes and 85 target lncRNAs that might be regulated by the 21 intersected DEMs among the 393 DEGs and the 116 intersecting DELs, respectively. The key lncRNA-miRNA and miRNA-mRNA pairs were shown in Supplementary Tables 3 and 4, respectively. According to the constructed association and the theory of ceRNAs, we chose the negatively correlated lncRNA-miRNA and miRNA-mRNA pairs to build an mRNA-miRNA-lncRNA network using Cytoscape v3.0 (Figure 3). As a result, 59 lncRNAs were identified as ceRNAs that interacted with 17 essential miRNAs, and subsequently indirectly regulated 63 coding mRNAs involved in HCC development.

Relying on the constructed relationships in the ceRNA network, we inferred that lncRNAs might indirectly participate in several significant KEGG pathways by serving as ceRNAs of mRNAs, such as the PI3K-AKT signaling pathway enriched by *ITGA2*, *PRKAA2*, and *CDK1*, the cell cycle enriched by *MCM5*, *CDC6*, and *CHEK1*, as well as ECM-receptor interactions enriched by *ITGA6* and *ITGA2*. Similarly, we also inferred that lncRNAs participated in some significant biological processes, including cell division enriched by *BIRC5* and *CDCA5*, as well as cell proliferation enriched by *CD34* and *ITGA2*. Indeed, some miRNA-mRNA and lncRNA-miRNA pairs in this network have been verified to play a part in cancer development in previous studies. For instance, Jafarzadeh et al*.* provided experimental evidences for hsa-miR-497-5p as a negative regulator of SMAD3, which was a key modulator of the TGF-β signaling pathway during carcinogenesis [21]. Among the genes interacting with hsa-miR-497-5p in this network, *BIRC5*, a well-known cancer-related gene encoding survivin, has been proven to be upregulated by TGF-β to modulate the cell cycle and apoptosis in various types of cancer [22, 23]. Therefore, the regulatory relationship between hsa-miR-497-5p and *BIRC5* presented in the ceRNA network was justifiable. Besides, for lncRNAs, *MCM3AP-AS1* has been reported to directly bind to miR-194-5p and act as ceRNA, which subsequently facilitated the expression of miR-194-5p’s target gene *FOXA1* in HCC cells, thus promoting HCC cell proliferation, colony formation, and cell cycle progression [24]. Another lncRNA identified in this network, *HAND2-AS1*, has been proven to increase cell migration of HCC cell lines [25].

### PPI (protein-protein interaction) network analysis

Subsequently, we built a protein-protein interaction network predicting the interaction among the proteins encoded by the 51 DEGs in the ceRNA network (Supplementary Figure 4). In the network, *ESR1*, *IGF1*, *BIRC5*, and *CD34* had higher degrees (17, 15, 12, and 10, respectively) (Supplementary Table 5). The genes encoding these proteins have been confirmed to be associated with HCC progression [26–29]. Combined with the functional analysis results, we found that the interacting DEGs in the ceRNA network were mainly enriched in cell proliferation, cell adhesion, the PI3K-Akt signaling pathway, the p53 signaling pathway, pathways in cancer, and metabolic pathways.

### Survival analysis and expression validation for the ceRNA network in TCGA dataset

**Figure 4:**
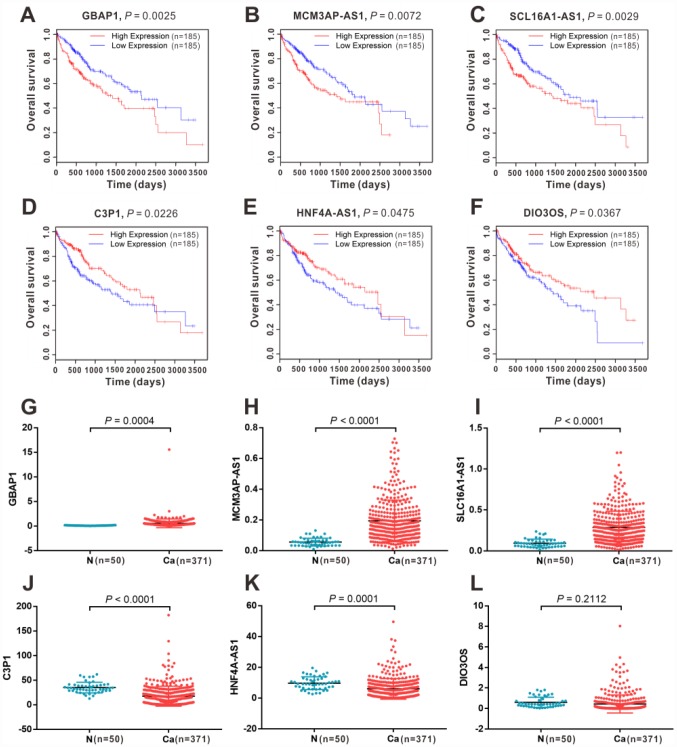
**The expression and survival significance of the six key lncRNAs in the ceRNA network in HCC TCGA database.** Kaplan-Meier survival curves showed significant OS differences between high- and low-expression of *GBAP1* (**A**), *MCM3AP-AS1* (**B**), *SLC16A1-AS1* (**C**), *C3P1*(**D**), *HNF4A-AS1* (**E**) and *DIO3OS* (**F**) in TCGA dtabase. Expression levels of *GBAP1* (**G**), *MCM3AP-AS1* (**H**), *SLC16A1-AS1* (**I**), *C3P1*(**D**), *HNF4A-AS1* (**E**) and *DIO3OS* (**F**) in normal and HCC tissues from TCGA database. N, normal tissue; Ca, cancer tissue.

In the Gene Expression Omnibus (GEO) datasets, we identified an extensive and comprehensive lncRNA-miRNA-mRNA regulation network in HCC development. To further clarify their expression and prognostic value, we performed Kaplan-Meier survival analysis for all the DEGs, DELs, and DEMs in the ceRNA network in patients with HCC from TCGA. The results showed that 6 lncRNAs (Figure 4), 23 mRNAs, and 4 miRNAs had a significant impact on OS (Supplementary Figure 5) and a consistent expression pattern (Supplementary Figure 6) in the GEO datasets. Among them, 3 lncRNAs (*GBAP1*, *MCM3AP-AS1*, and *SLC16A1-AS1*) (Figure 4A–4C, 4G–4I), 15 mRNAs (*ACLY*, *BAX*, *BIRC5*, *CDC6*, *CDCA5*, *CHEK1*, *FOXM1*, *ITGA2*, LAMC1, *MCM5*, *MYB*, *PAK1*, *PRKAA2*, *SOX4*, and *TK1*) (Supplementary Figures 5Aa–5Ao, 6Aa–6Ao) and 3 miRNAs (hsa-miR-10b-5p, hsa-miR-183-5p, and hsa-miR-222-3p) (Supplementary Figures 5Aa–Ao, 6Ba–Bc) were identified as pro-tumor factors because of their high expression in cancer tissues and their correlation with shorter OS in patients with HCC. In contrast, another three lncRNAs (*C3P1*, *DIO3OS*, *and HNF4A-AS1*) (Figure 4D–4F, 4J–4L), eight mRNAs (*ABAT*, *ACSM2A*, *ASPA*, *CAMK4*, *CYP8B1*, *ESR1*, *IGF1*, and *PDE7B*) (Supplementary Figure 5Ca–5Ch, 6Ca–6Ch) and one miRNA (hsa-let-7c-5p) (Supplementary Figures 5D, 6D) showed low expression in cancer tissues and correlated with longer OS, implying that these transcripts might be protective factors in HCC.

According to the interactions in the ceRNA network, we inferred that the aberrant expression of DEGs might be indirectly regulated by six lncRNAs (*GBAP1*, *MCM3AP-AS1*, *SLC16A1-AS1*, *C3P1*, *DIO3OS*, and *HNF4A-AS1*). To further determine the association of the identified lncRNAs with survival time, we conducted univariate and multivariate Cox regression model analyses based on the clinical characteristics in TCGA dataset. As shown in Table 2, in the univariate analysis, all six lncRNAs could be incorporated in the COX regression model. Multivariate analysis demonstrated that the expression levels of *lncRNAs C3P1*, *DIO3OS*, and *SLC16A1-AS1* were independent prognostic factors for OS in patients with HCC. Thus, it was reasonable to infer that these lncRNAs might be crucial factors to predict the prognosis of patients with HCC.

**Table 2:** Univariate and multivariate analysis of OS in 371 HCC patients from TCGA.

**Variable**	**Univariate cox**		**Multivariate cox**	
	***P*-value**	**HR(95%CI)**	***P*-value**	**HR(95%CI)**
**Age**				
≥50 or<50	0.649857	1.11(0.72-1.70)		
**Gender**				
Male or female	0.259970	1.23(0.86-1.75)		
**HBsAg**				
Positive or negative	**0.000018**	2.36(1.59-3.49)	**0.000310**	2.18(1.43-3.33)
**Liver cirrhosis**				
Yes or no	**0.002210**	0.54(0.37-0.80)	**0.030828**	0.64(0.42-0.96)
**TNM stage**				
III/IV or I/II	**0.000011**	2.23(1.56-3.19)	**0.002199**	1.80(1.24-2.62)
**AFP**				
≥400ng/ml or<400ng/ml	0.328566	0.79(0.50-1.26)		
**Histological differentiation**				
Poor or well	0.538730	1.12(0.78-1.61)		
**Vascular invasion**				
Yes or no	0.866489	1.03(0.70-1.52)		
**C3P1 expression**				
High or low	**0.023555**	0.67(0.47- 0.95)	**0.017161**	0.64(0.45-0.92)
**HNF4A-AS1 expression**				
High or low	**0.048667**	0.70(0.50-0.10)	0.123098	
**DIO3OS expression**				
High or low	**0.037804**	0.69(0.49-0.98)	**0.027333**	0.67(0.47-0.96)
**MCM3AP-AS1 expression**				
High or low	**0.007812**	1.61(1.13-2.28)	0.384940	
**GBAP1 expression**				
High or low	**0.003447**	1.68(1.19-2.38)	0.068636	1.40(0.97-2.01)
**SLC16A1-AS1 expression**				
High or low	**0.003193**	1.69(1.19-2.39)	**0.011557**	1.59(1.11-2.29)

### Correlation analysis in TCGA dataset

**Figure 5:**
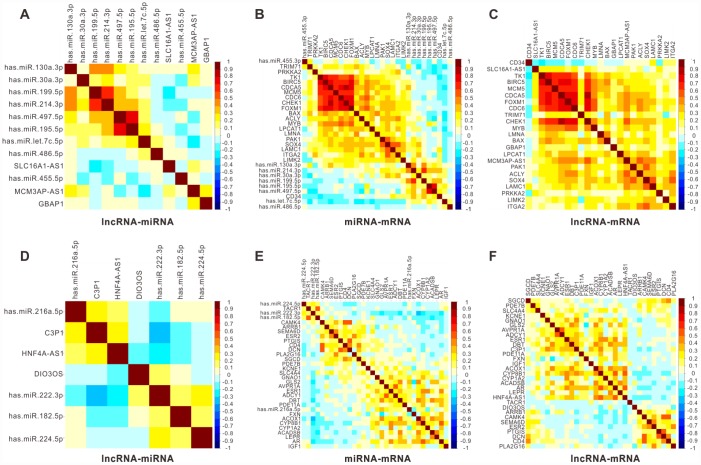
**Pearson correlation analysis for six key lncRNAs with their associated miRNA and mRNAs in 366 HCC patients from TCGA.** Pearson correlograms of lncRNA-miRNA paris (**A**), lncRNA-mRNA pairs (**B**) and mRNA-miRNA pairs (**C**) in upregulated lncRNAs including *GBAP1*, *MCM3AP-AS1* and *SLC16A1-AS1*. lncRNA-miRNA paris (**D**), lncRNA-mRNA paris (**E**) and mRNA-miRNA paris (**F**) in downregulated lncRNAs including *C3P1*, *HNF4A-AS1* and *DIO3OS* by Pearson correlation analysis. The correlation coefficient R value ranges from -1 to 1, the color of which changes from blue to brown.

Since the main aim of this study was identifying the clinical noteworthy lncRNAs and uncovering the regulatory role of lncRNAs as potential ceRNAs to mediate downstream/upstream RNAs functions/ associations, we selected the six lncRNAs with great survival significance in the ceRNA network for further study. To validate the regulatory role of the six lncRNAs as ceRNAs, we analyzed the correlation with their associated miRNAs and mRNAs in the ceRNA network based on TCGA data containing 366 patients with HCC. As shown in Figure 5, a considerable number of lncRNA-miRNA pairs showed a significant negative correlation for both upregulated (Figure 5A) and downregulated (Figure 5D) lncRNAs. Some of the six lncRNAs-associated miRNA-mRNA pairs also demonstrated a negative correlation (Figure 5B and 5E). Moreover, almost all six lncRNAs-associated lncRNA-mRNA pairs, for either upregulated (Figure 5C) or downregulated (Figure 5F) lncRNAs, demonstrated a tight positive correlation in TCGA database. Together, these results indicated the proposed six lncRNAs, *GBAP1*, *MCM3AP-AS1*, *SLC16A1-AS1 C3P1*, *DIO3OS*, and *HNF4A-AS1*, had a partially negative correlation with their associated miRNAs, as well as a partially positive correlation with their associated mRNAs, further confirming that the six might function as ceRNAs to regulate the expression of mRNAs and miRNAs in the progression of HCC.

### Expression validation and survival analysis for the six lncRNAs in clinical samples

**Figure 6:**
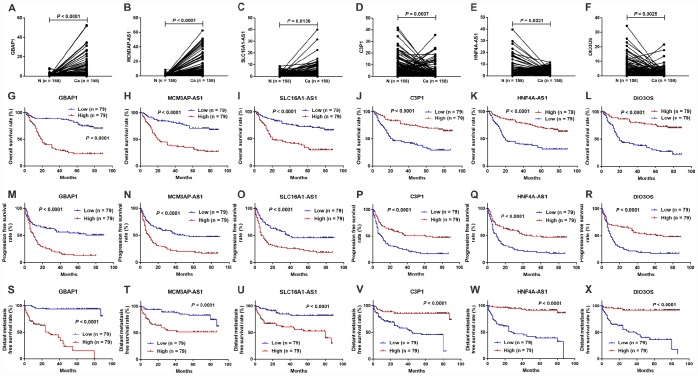
**Expression and survival sifnificance for the six lncRNAs in 158 clinical samples.** Expression levels of lncRNAs *GBAP1* (**A**), *MCM3AP-AS1* (**B**), *SLC16A1-AS1* (**C**), *C3P1* (**D**), *HNF4A-AS1* (**E**), and *DIO3OS* (**F**) in 158 HCC tissues and adjacent normal tissues. Kaplan-Meier survival curves showed significant OS differences between high- and low-expression of lncRNAs including *GBAP1* (**G**), *MCM3AP-AS1* (**H**), *SLC16A1-AS1* (**I**), *C3P1*(**J**), *HNF4A-AS1* (**K**), and *DIO3OS* (**L**), and significant PFS differences including *GBAP1* (**M**), *MCM3AP-AS1* (**N**), *SLC16A1-AS1* (**O**), *C3P1* (**P**), *HNF4A-AS1* (**Q**) and *DIO3OS* (**R**), as well as significant DmFS differences including *GBAP1* (**S**), *MCM3AP-AS1* (**T**), *SLC16A1-AS1* (**U**), *C3P1* (**V**), *HNF4A-AS1* (**W**) and *DIO3OS* (**X**). N =158, Ca = 158; N, normal tissue; Ca, cancer tissue.

To further verify the significance of the six lncRNAs mentioned in section 2.8, we analyzed their expression levels in tumor tissues and adjacent non-tumor tissues from 158 diagnosed patients with HCC using quantitative real-time reverse transcriptase-polymerase chain reaction (RT-PCR). As shown in Figure 6A–6F, all six lncRNAs were differentially expressed in tumor tissues and normal tissues. Three lncRNAs (*GBAP1*, *MCM3AP-AS*1, and *SLC16A1-AS1*) were upregulated in HCC tissues (Figure 6A–6C), while the other three lncRNAs (*C3P1*, *HNF4A-AS1*, and *DIO3OS*) were downregulated in HCC tissues (Figure 6D–6F). These results were consistent with the previous bioinformatic analysis. Furthermore, based on the clinical information, we analyzed the relationship between the expression levels of these DELs and OS, progression-free survival (PFS) and distant metastasis-free survival (DmFS) in these patients, which was demonstrated using Kaplan-Meier curves in Figure 6G–6X. Consistent with the results in TCGA, the upregulated lncRNAs, including *GBAP1*, *MCM3AP-AS1*, and *SLC16A1-AS1*, were associated with worse OS (Figure 6G–6I), PFS (Figure 6M–6O) and DmFS (S-U), thus identifying them as pro-tumor factors. Inversely, the downregulated lncRNAs, including *C3P1*, *HNF4A-AS1*, and *DIO3OS*, were correlated with the better OS (Figure 6J–6L), PFS (Figure 6P–6R) and DmFS (Figure 6V–6X), and thus represented protective factors. Importantly, combined with the clinical characteristics, we constructed univariate and multivariate Cox proportional hazards regression models for OS, PFS, and DmFS. As shown in Table 3, multivariate Cox’s regression analysis revealed that *DIO3OS*, *MCM3AP-AS*1, and *SLC16A1-AS1* expression levels were independent prognostic factors for OS. *C3P1*, *MCM3AP-AS1*, and *SLC16A1-AS1* expression levels were independent prognostic factors for PFS, and *HNF4A-AS1*, *MCM3AP-AS*1, *GBAP1*, and *SLC16A1-AS1* expression levels were independent prognostic factors for DmFS. These data indicated that the proposed six lncRNAs were clinical noteworthy biomarkers for predicting the prognosis and metastasis for patients with HCC.

**Table 3:** Univariate and multivariate analysis of OS and PFS in 158 HCC patients.

**Variable**	**Univariate cox**						**Multivariate cox**					
	**Overall survival**		**Progression free survival**		**Distant metastasis-freesurvival**		**Overall survival**		**Progression free survival**		**Distant metastasis-free survival**	
	***P*-value**	**HR (95%CI)**	***P*-value**	**HR (95%CI)**	***P*-value**	**HR (95%CI)**	***P*-value**	**HR (95%CI)**	***P*-value**	**HR (95%CI)**	***P*-value**	**HR (95%CI)**
**Age**												
≥50 or<50	0.8201	1.05 (0.67-1.66)	0.1448	1.34 (0.91-1.97)	0.6109	1.16 (0.65-2.06)						
**Gender**												
Male or female	0.6688	1.13 (0.64-1.99)	0.9348	1.02 (0.64-1.62)	0.7827	0.91 (0.46-1.79)						
**HBsAg**												
Positive or negative	0.6781	1.17 (0.56-2.43)	0.3082	1.39 (0.74-2.59)	0.7134	0.83 (0.30-2.30)						
**Liver cirrhosis**												
Yes or no	0.9817	1.01 (0.64-1.58)	0.3427	0.83 (0.56-1.23)	0.2657	0.72 (0.40-1.29)						
**Tumor size**												
≥5cm or<5cm	0.2149	0.75 (0.47-1.19)	0.1353	0.74 (0.49-1.10)	0.0723	0.57 (0.31-1.05)					**0.0422**	2.08 (1.03-4.23)
**TNM stage**												
III or I/II	**0.0306**	1.66 (1.05-2.64)	0.1796	1.32 (0.88-1.97)	**<0.0001**	9.06 (4.59-17.90)					**0.0009**	3.92 (1.75-8.80)
**Tumor encapsulation**												
Yes or no	**0.0021**	0.47 (0.29-0.76)	0.3202	0.82 (0.55-1.22)	0.0584	0.55 (0.30-1.02)						
**Tumor number**												
Multiple or single	**0.0009**	2.20 (1.38-3.52)	**0.0115**	1.66 (1.12-2.46)	0.1019	1.63 (0.91-2.93)	**0.0113**	1.90 (1.16-3.11)	**0.0495**	1.49 (1.00-2.23)		
**AFP**												
≥400ng/ml or <400ng/ml	0.3263	1.25 (0.80-1.97)	0.6073	1.11 (0.75-1.63)	0.0152	2.09 (1.15-3.79)						
**Histological differentiation**												
Poor or well	0.0923	1.77 (0.91-3.45)	0.1932	1.43 (0.83-2.49)	0.1072	2.15 (0.85-5.44)						
**Vascular invasion**												
Yes or no	0.0785	1.69 (0.94-3.01)	**0.0033**	2.09 (1.28-3.42)	**<0.0001**	5.97 (3.26-10.95)					**0.0100**	2.42 (1.24-4.75)
**C3P1 expression**												
High or low	**<0.0001**	0.32 (0.20-0.52)	**<0.0001**	0.43 (0.29-0.65)	**<0.0001**	0.20 (0.10-0.40)	0.1021	-	**0.0374**	0.61 (0.38-0.97)	0.5810	-
**HNF4A-AS1 expression**												
High or low	**<0.0001**	0.36 (0.23-0.58)	**0.0002**	0.47 (0.32-0.69)	**<0.0001**	0.08 (0.04-0.18)	0.4931	-	0.9147	-	**0.0052**	0.30 (0.13-0.70)
**DIO3OS expression**												
High or low	**<0.0001**	0.16 (0.09-0.28)	**<0.0001**	0.34 (0.22-0.52)	**<0.0001**	0.17 (0.08-0.35)	**0.0042**	2.14 (1.27-3.61)	0.0865	0.64 (0.38-1.07)	0.3061	-
**MCM3AP-AS1 expression**												
High or low	**<0.0001**	3.75 (2.27-6.17)	**<0.0001**	2.38 (1.59-3.55)	**<0.0001**	3.54 (1.88-6.64)	**0.0042**	2.14 (1.27-3.61)	**0.0092**	1.74 (1.15-2.65)	**0.0092**	2.77 (1.29-5.98)
**GBAP1 expression**												
High or low	**<0.0001**	5.31 (3.14-8.99)	**<0.0001**	2.67 (1.77-4.02)	**<0.0001**	23.38 (8.74-62.56)	0.0625	-	0.4362	-	**0.0005**	6.83 (2.32-20.12)
**SLC16A1-AS1 expression**												
High or low	**<0.0001**	3.75 (2.27-6.17)	**<0.0001**	2.38 (1.59-3.55)	**<0.0001**	3.54 (1.88-6.64)	**0.0001**	2.79 (1.65-4.70)	**0.0053**	1.82 (1.19-2.78)	**0.0156**	2.36 (1.18-4.73)

### Correlation validation for the six lncRNAs in clinical samples

**Figure 7:**
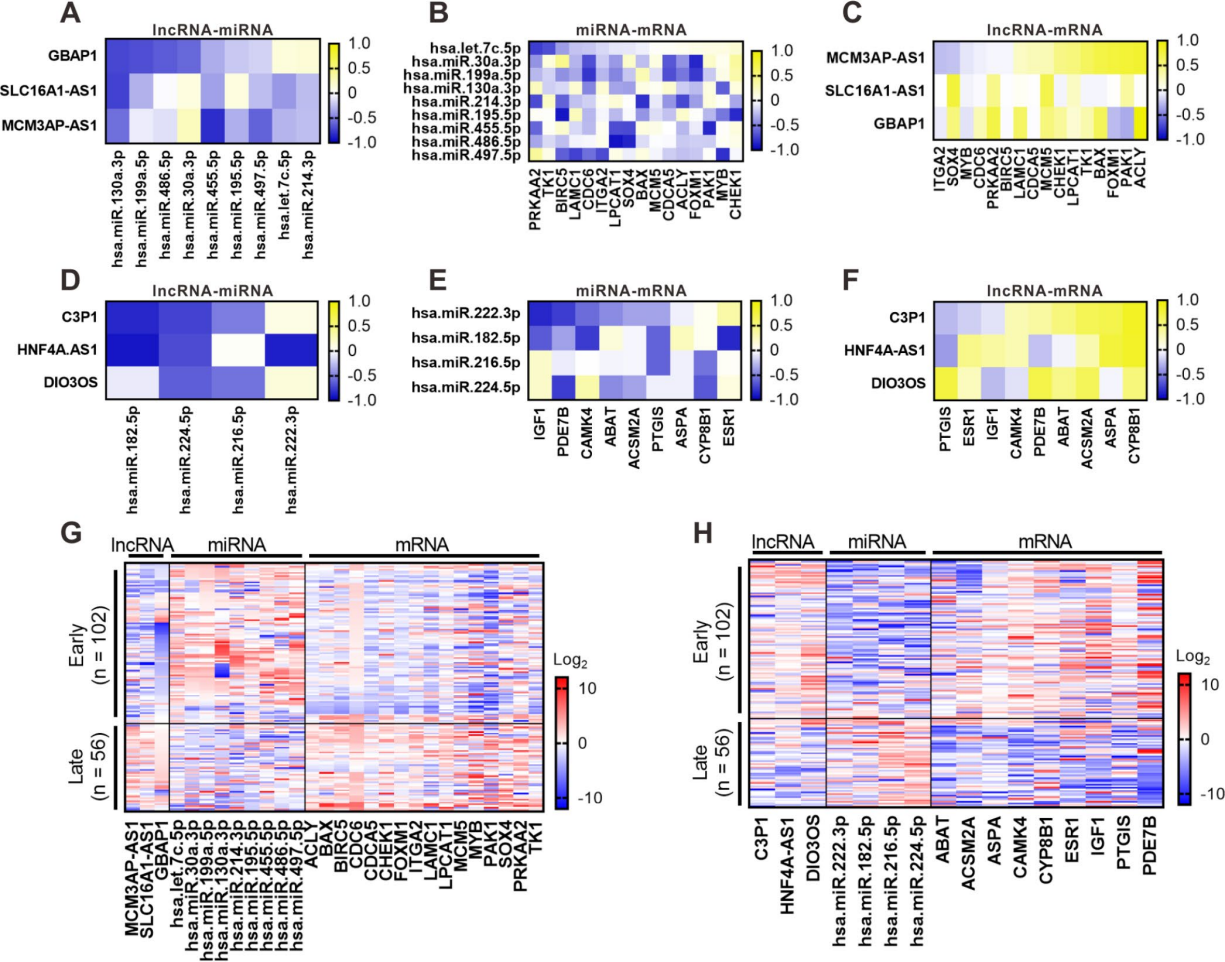
**Correlation validation for the six lncRNAs with their associated miRNA and mRNAs in 158 clinical samples of HCC.** RT-PCR was performed to detect the expression of the six lncRNAs associated miRNA and mRNAs in 158 clinical samples. Pearson correlation analysis for six key lncRNAs with their associated miRNA and mRNAs was performed based on RT-PCR results. Pearson correlograms of lncRNA-miRNA paris (**A**), lncRNA-mRNA pairs (**B**) and mRNA-miRNA pairs (**C**) in upregulated lncRNAs including *GBAP1*, *MCM3AP-AS1* and *SLC16A1-AS1*. lncRNA-miRNA paris (**D**), lncRNA-mRNA paris (**E**) and mRNA-miRNA paris (**F**) in downregulated lncRNAs including *C3P1*, *HNF4A-AS1* and *DIO3OS* by Pearson correlation analysis. The correlation coefficient R value ranges from -1 to 1, the color of which changes from blue to yellow. 158 clinical samples were diveded into early and late groups according to TMN stage (early = TNM I or II; late = III), the expression of the six lncRNAs associated miRNA and mRNAs was shown as heatmaps. (**G**) Expression of the upregulated lncRNA *GBAP1*, *MCM3AP-AS1* and *SLC16A1-AS1* and their ssociated miRNA and mRNAs. (**H**) Expression of th edownregulated lncRNA *C3P1*, *DIO3OS*, and *HNF4A-AS1* and their ssociated miRNA and mRNAs. Log_2_(P) value was shown as color ranging from blue to red.

To further understand the links of the six lncRNAs with miRNAs or mRNAs in the ceRNA network, we selected lncRNAs associated miRNAs, as well as specific mRNAs with survival significance in TCGA dataset of HCC from the ceRNA network. And their expression levels were assessed by RT-PCR in 158 clinical samples. By correlation analysis based on RT-PCR results, a significantly negative correlation was found between a majority of lncRNA-miRNA pairs for both upregulated (Figure 7A) and downregulated (Figure 7D) lncRNAs. In accordance with TCGA dataset, most of the lncRNAs-associated miRNA-mRNA pairs demonstrated a negative correlation in clinical samples (Figure 7B and 7E), and the positive correlation in a great part of lncRNA-mRNA pairs was also observed (Figure 7C and 7F). Furthermore, we analyzed the expression pattern of the proposed ceRNA network in samples from different stages (early = TNM I or II; late = III) based on the results of RT-PCR, including the six lncRNAs and associated miRNAs and specific mRNAs. As shown in Figure 7G, we found the upregulated lncRNA *GBAP1*, *MCM3AP-AS1* and *SLC16A1-AS1* were highly expressed in late-stage samples compared with early-stage samples. Inverse and similar expression patterns were observed in their associated miRNAs and mRNAs, respectively (Figure 7G). In contrast, the downregulated lncRNA *C3P1*, *DIO3OS*, and *HNF4A-AS1* were low expressed in late-stage samples compared to early-stage samples (Figure 7H). Inverse and similar expression patterns were also observed in their associated miRNAs and mRNAs, respectively (Figure 7H). Taken together, these data further verified that the six lncRNAs might play an important role as ceRNAs in regulating the expression of miRNAs and mRNAs. Moreover, upregulation of *GBAP1*, *MCM3AP-AS1*, and *SLC16A1-AS1* and downregulation of *C3P1*, *DIO3OS*, and *HNF4A-AS1* might be involved in regulating the progression of HCC from early to late stage.

### Receiver operating characteristic (ROC) curves analysis for the key lncRNAs in clinical samples

**Figure 8:**
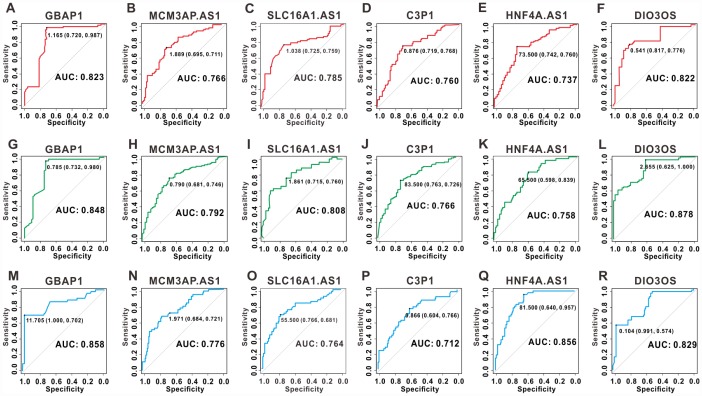
**ROC curves for the six key lncRNAs in 158 clinical samples.** ROC curves of lncRNA *GBAP1*, *MCM3AP-AS1*, *SLC16A1-AS1*, *C3P1*, *HNF4A-AS1* and *DIO3OS* for OS (**A–F**), PFS (**G–L**), and DmFS (**M–R**) respectively. The AUCs under binomial exact confidence interval was calculated to generate the ROC curve.

To assess the discriminatory ability of the six key lncRNAs in HCC, ROC curve analyses were conducted in 158 patients with HCC and the areas under the curve (AUCs) were calculated. As shown in Figure 8A–8F, in the assessment of OS, the AUCs of five lncRNAs (*GBAP1*, *MCM3AP-AS1*, *SLC16A1-AS1*, *C3P1*, and *DIO3OS*) were more than 0.75, and the AUCs of *HNF4A-AS1* was 0.737. In the assessment of PFS, the AUCs of all the six lncRNAs were greater than 0.75 (Figure 8G–8L). Additionally, five lncRNAs (*GBAP1*, *MCM3AP-AS1*, *SLC16A1-AS1*, *HNF4A-AS1* and *DIO3OS*) had a good performance in diagnosing DmFS with AUCs above 0.75, and the AUCs of *C3P1* was 0.712 (Figure 8M–8R). These results suggested that the six lncRNAs had good sensitivity and specificity to predict survival and distant metastasis in patients with HCC.

## DISCUSSION

In recent decades, the diagnosis and treatment of HCC have improved dramatically. Although patients with HCC have benefited from multiple options that improve their survival, regardless of the cancer stage at diagnosis, the survival time of patients with HCC remains limited [30]. Apart from traditional surgical resection, ablation, and systematic chemotherapy, the targeted molecular therapy has become a promising therapeutic option for patients with advanced-stage disease or patients who transitioned into it after other therapies failed [1, 2]. However, the efficacy of this improved therapy is still limited to certain patients. Thus, there is an urgent need to discover more molecules that drive HCC tumorigenesis and progression to develop new and effective therapeutic targets for HCC.

Non-coding RNAs have raised considerable research interest due to their regulation of the transcription of protein-coding genes to accelerate cancer progression. As typical non-coding RNAs, lncRNAs and miRNAs have been extensively implicated in the oncogenesis of a variety of cancers [31], including HCC [14, 15]. In the present study, we firstly analyzed five expression profiles to identify differentially expressed genes, lncRNAs, and miRNAs between HCC samples and normal samples based on data from the GEO database. For the identified DEGs, GO functional enrichment analysis showed that the upregulated DEGs were most significantly associated with cell division, a process that allows cells to proliferate persistently, while the downregulated DEGs were mainly involved in the oxidation-reduction process. Consistently, it has been reported that the oxidant production, such as H_2_O_2_, is elevated in the tumor microenvironment because of the imbalance between oxidation and reduction. Some oxidants can trigger cell growth and immune cell dysfunction in different kinds of cancer types, including HCC [32–34]. KEGG pathway analysis demonstrated that the upregulated DEGs were most strongly implicated in the cell cycle, and downregulated DEGs mainly participated in metabolic pathways. Abnormalities of these pathways were considered as two essential hallmarks of cancer [35]. The signal-net analysis demonstrated how these DEGs influenced each other, which suggested that some hub genes exerted central regulatory functions, such as *PRKAA2*, *ITGB3*, *PLCB1*, and *ITGA2*, providing further scientific clues to study and treat HCC. Consistent with our results, Zhang et al*.* confirmed that the upregulated expression of *ITGB3* mediated the expression of *MMP2* by activating the FAK/PI3K/AKT signaling pathway, contributing to the enhancement of metastatic potential of residual cancer in the HCCLM3 model after insufficient radiofrequency ablation [36]. Similarly, *ITGB3* and *PLCB1* were also confirmed to facilitate HCC progression by enhancing the adhesion and proliferation of tumor cells. However, contradictory results were observed in some other cancer types, such as gastric and prostate cancer, which indicated that *PRKAA2* was a protective gene in cancer development by upregulating the expression of hypoxia-inducible factor-1α and hepatocyte nuclear factor 4α [37, 38]. More experimental evidence is required to verify the role of *PRKAA2* in the development of HCC.

MiRNAs mediate the expression of transcripts by binding to MREs of their target mRNAs. CeRNAs are a group of non-coding transcripts that maintain the balance between miRNAs and their target genes [10]. These targets include pseudogenes, protein-coding genes, and lncRNAs. The ceRNA hypothesis proposes that these transcripts can act as bona fide miRNA competitors owing to the presence of MREs or high-sequence homology. Therefore, ceRNAs actively compete with their ancestral protein-coding genes for the same pool of miRNAs. The consequence of competition for miRNAs is a decrease in their activity to its targets. Therefore, except for the conventional miRNA-RNA regulation, a reversed RNA-miRNA regulation relationship also exists, in which coding and noncoding RNA targets can exert crosstalk through their ability to compete for miRNA binding [10, 39, 40]. In this study, we constructed an mRNA-miRNA-lncRNA interaction network (ceRNA network) to show the interactive regulation relationships among the DEGs, DELs, and DEMs.

In this network, lncRNAs functioned as ceRNAs to sequester miRNAs and regulate mRNA transcripts containing shared MREs. The correlation analysis demonstrated a significant negative correlation in a majority of lncRNA-miRNA and miRNA-mRNA pairs, in line with the theoretical silencing effect between miRNAs and its target transcripts. Hence, the constructed mRNA-miRNA-lncRNA interaction network was a ceRNA crosstalk network. It reminded us of the potential regulatory mechanisms for transcripts involved in HCC development. Indeed, accumulating evidence pointed to the function of ceRNA network in regulating tumor progression. LncRNA *PVT1* significantly promoted autophagy and subsequent proliferation of tumor cells through acting as a ceRNA to target *ATG3* by sponging microRNA-365 in HCC [41]. Also, lncRNA *FAL1* was found to promote the proliferation and migration of HCC cells by acting as a ceRNA of miR-1236 [42]. Similarly, ceRNA networks also serve as important regulatory mechanisms to accelerate cancer progression in other cancer types. For example, lncRNA *PTAR* promoted EMT and invasion- metastasis in serous ovarian cancer by competitively binding to miR-101-3p to regulate *ZEB1* expression [12]. LncRNA-*KRTAP5-AS1* and lncRNA-*TUBB2A* could act as ceRNAs to affect the function of Claudin-4, which reinforces proliferation, invasion, and EMT in gastric cancer [43].

We also validated our results in TCGA data and clinical samples. Using survival analysis, COX regression analysis, and ROC analysis, we demonstrated that a proportion of DELs, DEMs, and DEGs in the ceRNA network had a significant impact on the OS of patients with HCC, presenting promising clinical and translational significance. Among them, *BAX*, *BIRC5*, *ITGA2*, and *MYB* were identified as significantly correlated with the survival of patients with HCC, which was in line with the results of previous studies [28, 44–46], while *PRKAA2*, *PTGIS*, *PDE7B*, *CYP8B1*, *CHEK1*, *CAMK4*, *ASPA*, and *ACSM2A* were identified to predict the prognosis of patients with HCC for the first time. Also, we found six lncRNAs and five miRNAs that might be valuable predictive factors for the survival of patients with HCC in both TCGA and clinical samples. LncRNA *MCM3AP-AS1* has shown the same result in the survival analysis in another group containing 80 HCC patients, which was also verified to facilitate the proliferation and suppress the apoptosis of HCC cells by acting as ceRNA of miR-194-5p targeting *FOXA1* [37]. Notably, the other five lncRNAs (*C3P1*, *DIO3OS*, *GBAP1*, *SLC16A1-AS1*, and *HNF4A-AS1*) and all five miRNAs were identified to be related to the prognosis of patients with HCC for the first time. Also, the results of correlation validation of the six lncRNAs with their associated miRNAs and mRNAs in TCGA were mostly consistent with the results in the constructed ceRNA network. This indicated that the network was reliable and that the six identified lncRNAs might play a role in the mechanisms of HCC progression. Therefore, they might have promising prognostic and therapeutic values in patients with HCC. However, there are also a few limitations of our study. For example, we demonstrated their relationship in the ceRNA network at the RNA level but in-depth work will be needed to verify their function using experimental data. Moreover, the data used were obtained from the GEO database, rather than directly from patients with HCC. Therefore, we need to perform a series of verification studies in a large-scale cohort of patients and at multiple centers to confirm these results.

In conclusion, the present study performed large-scale analyses and highlighted the complex crosstalk involving lncRNA-miRNA-mRNA networks and the important roles of lncRNAs as ceRNAs in HCC development. We identified a cluster of lncRNAs (*GBAP1*, *MCM3AP-AS1*, *SLC16A1-AS1*, *C3P1*, *DIO3OS*, and *HNF4A-AS1*) as potential ceRNAs that regulate HCC carcinogenesis and progression, defining them as specific biomarkers to diagnose HCC and predict the prognosis and metastasis of patients with HCC. These findings will improve our understanding of the ceRNAs’ regulatory mechanisms in HCC development and contribute to the identification of potential targets for the clinical diagnosis and treatment of patients with HCC.

## MATERIALS AND METHODS

### Patients and samples

Tissue samples, including HCC tumor tissues and adjacent non-cancerous tissues (n = 158), were obtained from patients who had resection of primary HCC in the Cancer Center of Sun Yat-sen University between 2005 and 2008. None of these patients had received preoperative chemotherapy or radiotherapy. After resection, matched fresh tissues were immersed immediately in RNAlater® (Ambion, Austin, TX, USA), kept overnight at 4 °C, and then stored at −80 °C until RNA isolation before qRT-PCR detection. Follow-up was performed in our outpatient department and involved clinical and laboratory examinations every three months for the first two years, every six months during the third to fifth years, and annually for an additional five years or until death, whichever occurred first. Follow-up periods for survivors ranged from 2 to 87 months, with a median follow-up of 41 months. OS and PFS were used as measures of prognosis. Written informed consent was obtained from each patient, and the Ethics Committee of Sun Yat-Sen University Cancer Center approved the study protocol.

### GEO gene expression datasets

HCC gene expression data were obtained from the GEO database (http://www.ncbi.nlm.nih.gov/geo/), including GSE29721, GSE40367, and GSE62232. The three profiles included a total of 120 samples, consisting of 98 cancerous and 22 normal samples. The mRNA expression profiles were obtained using an Affymetrix Human Genome U133 Plus 2.0 Array. According to the annotation file, we chose the probe sets and BLAST searched them in the NCBI RefSeq database to identify the probe sets of noncoding RNAs with a length of over 200 bp. We then used these probe sets to acquire the expression data of lncRNAs from the three profiles. The miRNA expression data were also obtained from the GEO databases. Two profiles, GSE36915 and GSE74618, were selected that contained 286 cancerous and 31 normal samples.

### Differential and clustering analysis

Based on the expression data from the datasets, a random variance model (RVM) t-test was applied to filter the DEGs, DELs, and DEMs among cancerous and normal samples. After significance analysis and FDR analysis, we selected the DEGs, and DELs from GSE29721, GSE40367, and GSE62232 according to the *P*-value threshold. *P* < 0.05 and FC > 2 was considered as significant difference. DEMs were selected using a threshold of *P*-value < 0.05, FDR < 0.05, and FC > 1.5. Hierarchical cluster analysis was performed and a cluster dendrogram was constructed to demonstrate distinct characterizations of screened DEGs, DELs, and DEMs between the cancerous and normal tissues. Furthermore, we selected the intersecting DEGs, DELs, and DEMs among the different profiles.

### GO and KEGG pathway analysis

Gene functional enrichment analysis was used to predict the biological functions of the intersected DEGs according to the GO database with *P* < 0.05 and FDR <0.05. The potentially involved signaling pathways were identified using the KEGG pathways analysis program (http://www.genome.jp/kegg/tool/map_pathway1.html)).

Two-side Fisher’s exact test and a *χ^2^* test were used to classify the GO categories, and the FDR [47] was calculated to correct the *P*-value: The smaller the FDR, the smaller the error in judging the *P*-value. The FDR was defined as:

$$\text{FDR}=\text{1}-N_{k}/T,$$ (1)

where *N_k_* referred to the number of Fisher’s test *P*-values less than the test *P*-values. We computed *P*-values for the GO categories of all the DEGs. Within the significant categories, the enrichment, Re, was calculated as:

$$\text{Re}=\left(n_{f}\text{/}n\right)\text{/}(N_{f}\text{/}N),$$ (2)

where “n_f_” was the number of flagged genes within the particular category, “*n*” was the total number of genes within the same category, “*N_f_*” was the number of flagged genes in the entire microarray, and “*N*” was the total number of genes in the microarray [48]. For KEGG pathway analysis, we again used Fisher’s exact test and *χ^2^* test to select the significant pathways, and the threshold of significance was defined *P*-value <0.05 and FDR < 0.05. The Re value was calculated using equation (2) [49- 51].

### Signal-net analysis of intersecting DEGs

Based on the KEGG database analysis, a gene-gene interaction network of DEGs was constructed to demonstrate the regulatory relationships among the DEGs identified in the intersection analysis among different expression profiles of mRNAs. The networks were stored and presented as graphs, where nodes were mainly genes (or proteins or compounds) and edges represented the type relationships between the nodes, e.g. activation or phosphorylation. We investigated the nature of networks using tools implemented in the R software.

The important nodes were identified computationally. To this end, we used the connectivity (also known as degree), which was defined as the sum of connection strengths with the other network genes:

$${\text{K}}_{i}=\sum_{u\neq i}a_{ui}$$, (3)

In the gene networks, the connectivity measured how a gene correlated with all other network genes. For a gene in the network, the number of source genes of a gene was called the indegree of the gene and the number of target genes of a gene was its outdegree. The character of a gene was described using betweenness centrality measures reflecting the importance of a node in a graph relative to other nodes. For a graph G: (V, E) with n vertices, the relative betweenness centrality ${C^{\prime }}_{B}(v)$ is defined by:

$${C^{\prime }}_{B}(v)=\frac{2}{n^{2}-3n+2}\sum_{\begin{array}{l} s\neq v\neq t\in V\\ s\neq t \end{array}}\frac{\sigma_{st}(v)}{\sigma_{st}},$$ (4)

where $\sigma_{st}$ is the number of shortest paths from s to t, and $\sigma_{st}(v)$ is the number of shortest paths from s to t that pass through a vertex v [52-56].

### Target transcripts of DEMs prediction and ceRNA network construction

Based on the functional DEGs identified in the GO and KEGG pathway analysis, we constructed the intersection datasets between the DEGs involved in the significant enriched GO terms and pathways with *P* < 0.05 and FDR < 0.05. Ultimately, 393 DEGs were selected, comprising 78 upregulated DEGs and 315 downregulated DEGs. Combining the intersecting DELs and DEMs, we predicted the targeted sponge lncRNAs using the miRanda tool (http://www.microrna.org/ microrna/home.do), and the target mRNAs using Targetscan (http://www.targetscan.org/) and miRWalk (http://129.206.7.150/). For each pair of miRNA-mRNA or mirRNA-lncRNA, we conducted Pearson correlation analysis and chose the significantly correlated pairs [57]. In particular, we summarized the intersecting targeted mRNAs discerned using miRanda and miRWalk.

Then, we chose the miRNAs that negatively regulated the expression levels of lncRNAs and mRNAs to construct a ceRNA network according to the normalized signal intensity of the expression of specific mRNAs and lncRNAs.

### Protein regulation network analysis

The STRING online database tool (https://string-db.org/cgi/input.pl) was used to construct a PPI network of the DEGs identified in the ceRNA network. The interacting pairs with a confidence score greater than 0.4 were considered as significant and were retained. The proteins encoded by the hub genes were screened according to the degree of the nodes. The degree represents the number of interaction partners and was calculated using Perl code.

### Survival analysis

We screened the HCC RNA-Seq TCGA datasets containing survival information and selected 370 patients with HCC as a dataset to analyze the relationship between the expression level of lncRNAs and mRNAs and OS. Another dataset that contained 371 patients with HCC was selected to analyze the relationship between the expression level of miRNAs and OS. In the clinical samples, we also investigated the correlation of lncRNAs with OS and PFS for 158 patients with HCC. According to the expression level of mRNAs, lncRNAs, and miRNAs in TCGA or our clinical samples, we classified them into two groups: High expression and low expression. Survival curves were displayed using Kaplan–Meier plots. The Wilcoxon log-rank test was used to analyze the survival difference between the high and low groups. All survival analyses in TCGA were conducted using the R package, Survival.

### Correlation analysis

To verify the correlation of expression among the miRNAs, lncRNAs, and mRNAs identified in the ceRNA network, we chose the lncRNAs that had significance for the prognosis of patients with HCC and their associated-mRNAs and miRNAs. Pearson correlation analyses were performed among them based on the RNA-Seq data of 366 patients with HCC searched in TCGA database (https://cancergenome.nih.gov/). The correlogram was constructed using the R package, Corrplot.

### RNA extraction and qRT-PCR

Total RNA was isolated from tissues using the TRIzol reagent (Invitrogen Corporation, Waltham, MA, USA) according to the manufacturer’s instructions. The concentration and purity of the RNA were evaluated using a NanoDrop 2000 instrument (Thermo Scientific, Waltham, MA, USA). For mRNA, the first-strand cDNA was synthesized from total RNA using a GoScript Reverse Transcription System (Promega, Madison, WI, USA). *GAPDH* was used as an endogenous control for normalization. For miRNAs, the first-strand cDNA was synthesized from total RNA using a riboSCRIPT^TM^ Reverse Transcription Kit (RIBOBIO, GuangzZhou, China). *U6* was used as an endogenous control for normalization. QPCR was performed using GoTaq qPCR Master Mix (Promega).

### ROC curve analysis

ROC analyses were performed using the pROC package in the R language, based on data from 158 clinical samples. The diagnostic ability of the prediction model was evaluated by calculating the area under a ROC curve. The ROC curve was used for classifier evaluation and was drawn by plotting sensitivity against the false-positive rate. The AUC under a binomial exact confidence interval was calculated to generate the ROC curve.

### Statistical analysis

Data are shown as the mean ± s.d. and analyzed using Student’s t-test. A paired t-test was used for paired samples. Statistical analyses were performed using GraphPad Prism 7 and SPSS software (GraphPad Software, La Jolla, CA, USA). A value of *P*<0.05 was considered statistically significant.

## Supplementary Material

Supplementary Figures

Supplementary Tables
