# Supplementary material for: Large-scale analyses identify a cluster of novel long noncoding RNAs as potential competitive endogenous RNAs in progression of hepatocellular carcinoma
Source: Aging (Albany NY). 2019 Nov 23;11(22):10422–53. doi: 10.18632/aging.102468 (PMC6914412; doi:10.18632/aging.102468)
Supplement: Supplementary Figures [file aging-11-102468-s002..pdf]

## SUPPLEMENTARY FIGURES

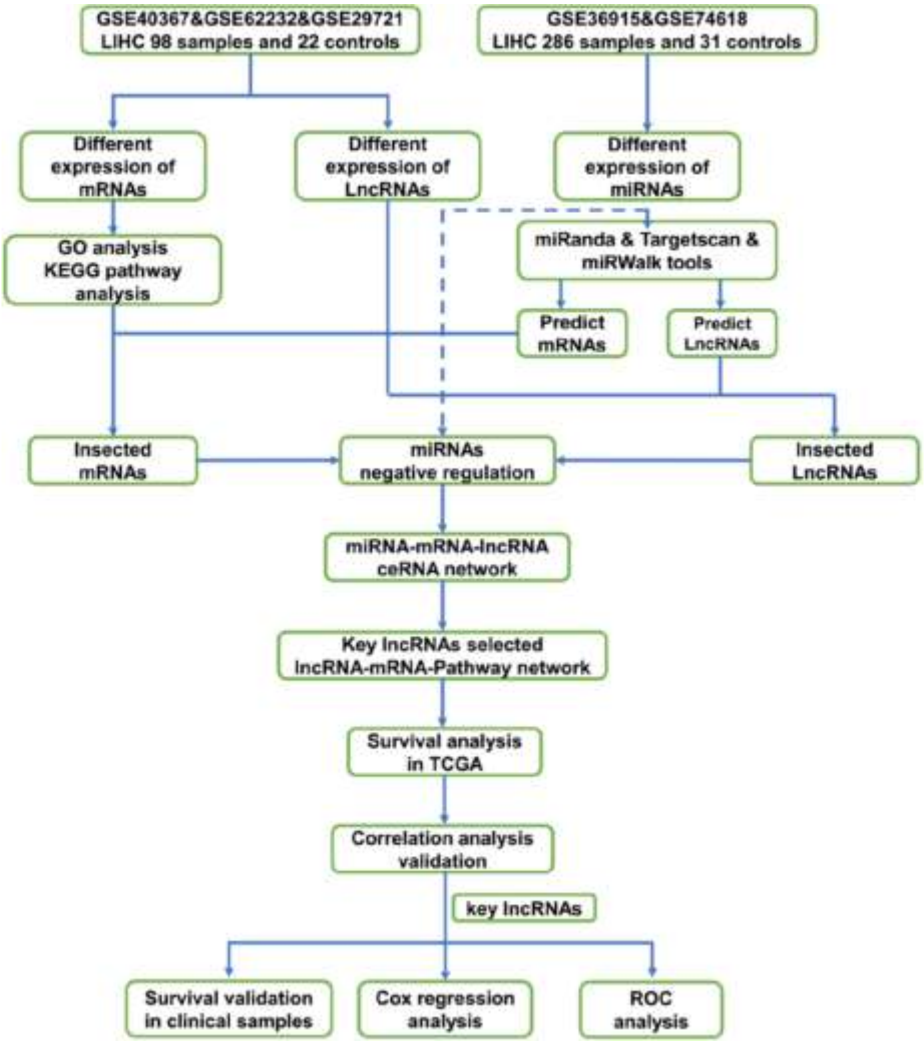


**Supplementary Figure 1. Bioinformatics analysis flowchart.**

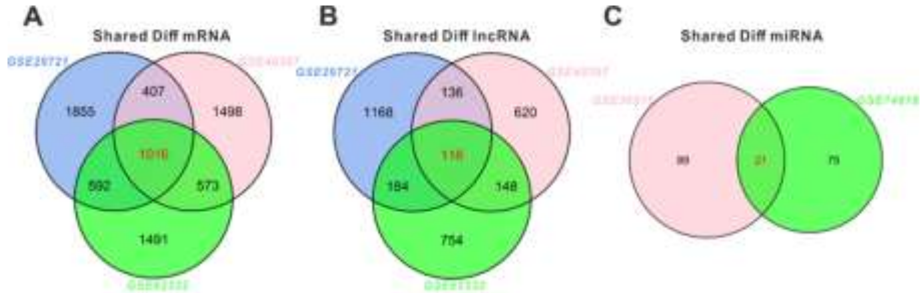


**Supplementary Figure 2. Venn diagram analysis of the intersected DEGs, DELs and DEMs among different expression profiles.** (**A**) The 1016 intersected DEGs obtained from GSE29721, GSE40367 and GSE62232. (**B**) The 116 intersected DELs obtained from GSE29721, GSE40367 and GSE62232. (**C**) The 21 intersected DEMs obtained from GSE36915 and GSE74618. Diff, Different.

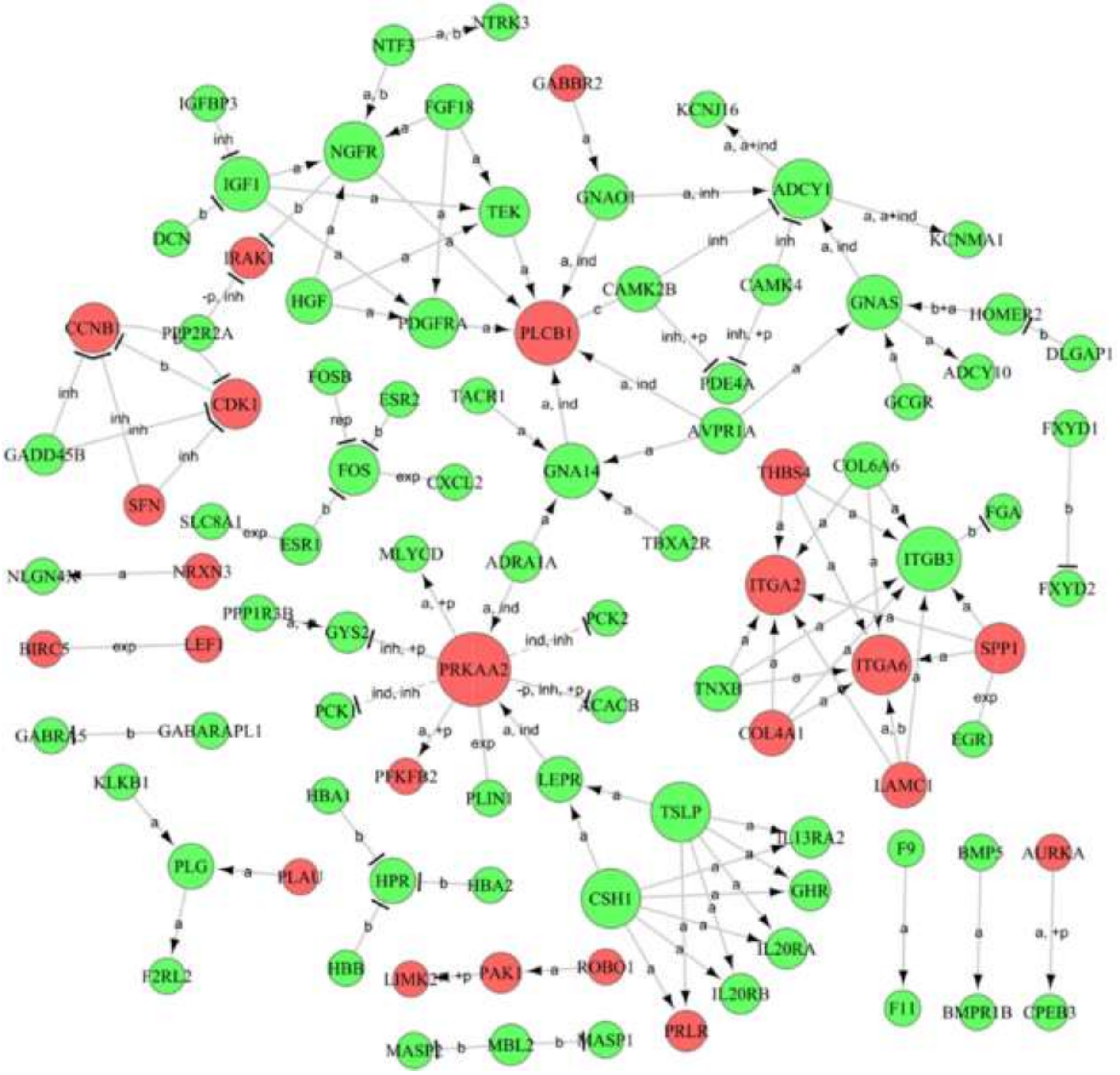


**Supplementary Figure 3. Signal network of differentially expressed mRNAs genes.** Red cycle nodes represent upregulated genes, and green cycle nodes represent downregulated genes. Edges represent interactions between genes (arrowheads represent targets). Interaction types: a, activation; b, binding/association; c, compound; exp, expression; ind, indirect effect; inh, inhibition; +p, phosphorylation; -p, dephosphorylation.

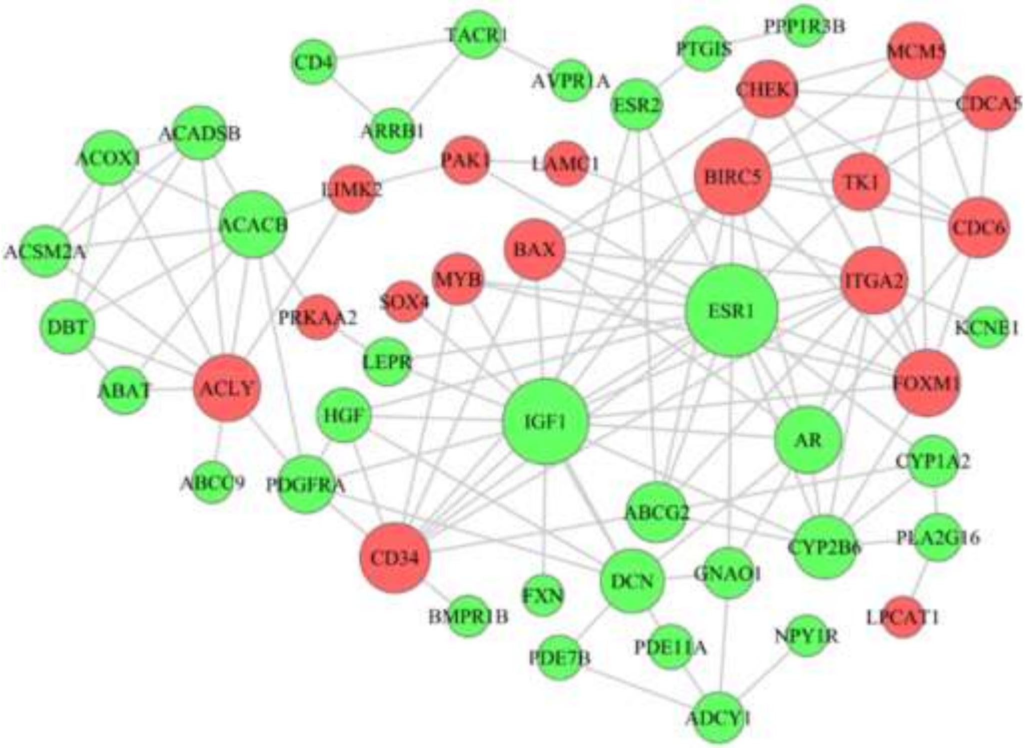


**Supplementary Figure 4. PPI network contrasted by the differentially expressed genes identified in the ceRNA network.** Red dots represent up-regulated genes and green dots represent down-regulated genes. The degree of the dots represents the ability to interact with other genes.

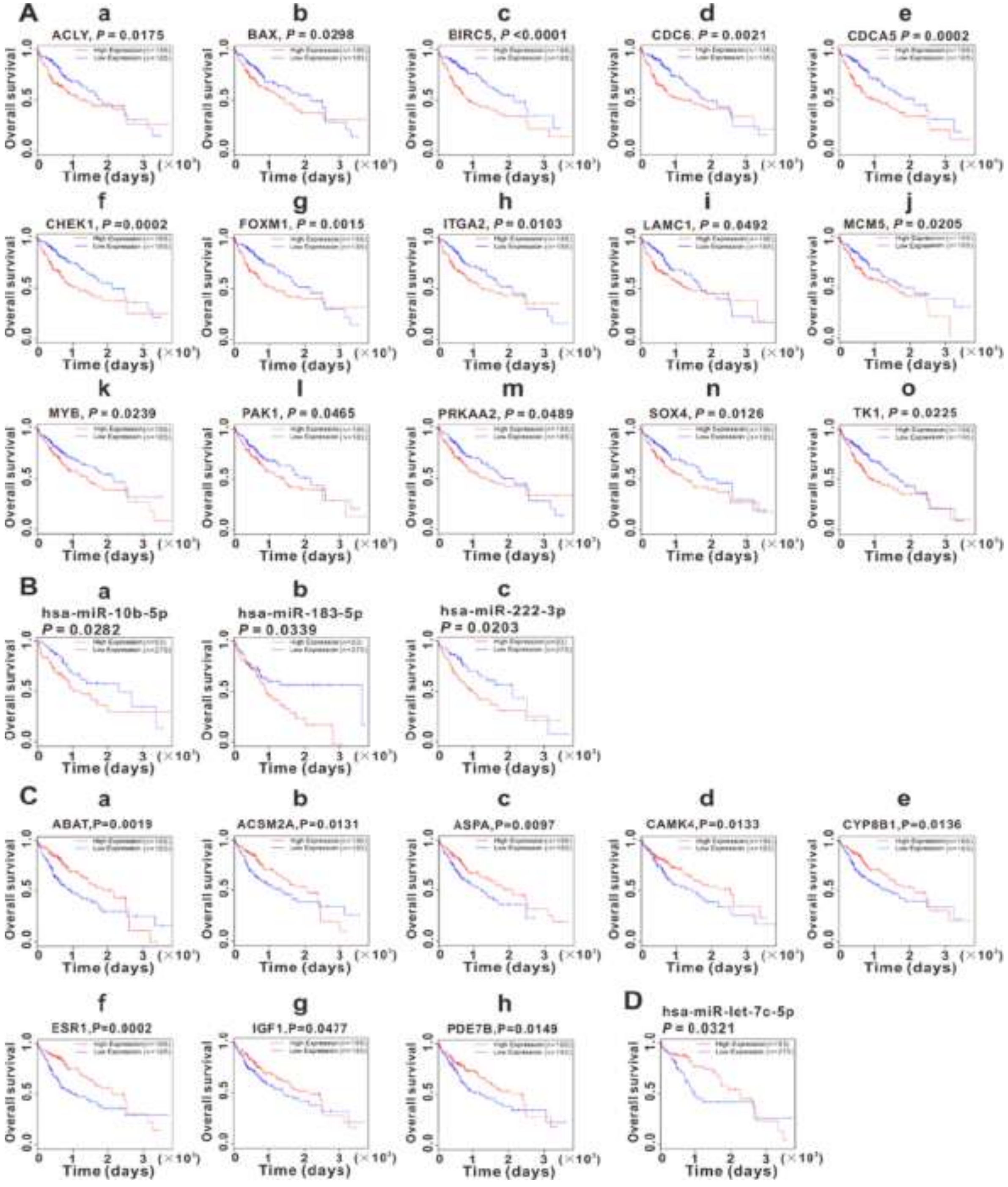


**Supplementary Figure 5. Survival significance of the DEGs and DEMs in ceRNA network in HCC TCGA database.** The Kaplan-Meier survival curves showed the significant correlation of DEGs and DEMs with OS time in HCC patients. The upregulated DEGs suggesting shorter OS were ACLY, BAX, BIRC5, CDC6, CDCA5, CHEK1, FOXM1, ITGA2, LAMC1, MCM5, MYB, PAK1, PRKAA2, SOX4, TK1 (**Aa**–**Ao**). The upregulated DEMs suggesting shorter OS were hsa-miR-10b-5p, hsa-miR-183-5p, and hsa-miR-222-3p (**Ba**–**Bc**). The upregulated DEGs suggesting longer OS were ABAT, ACSM2A, ASPA, CAMK4, CYP8B1, ESR1, IGF1, and PDE7B (**Ca**–**Ch**). The upregulated DEMs suggesting longer OS were hsa-let-7c-5p (**D**).

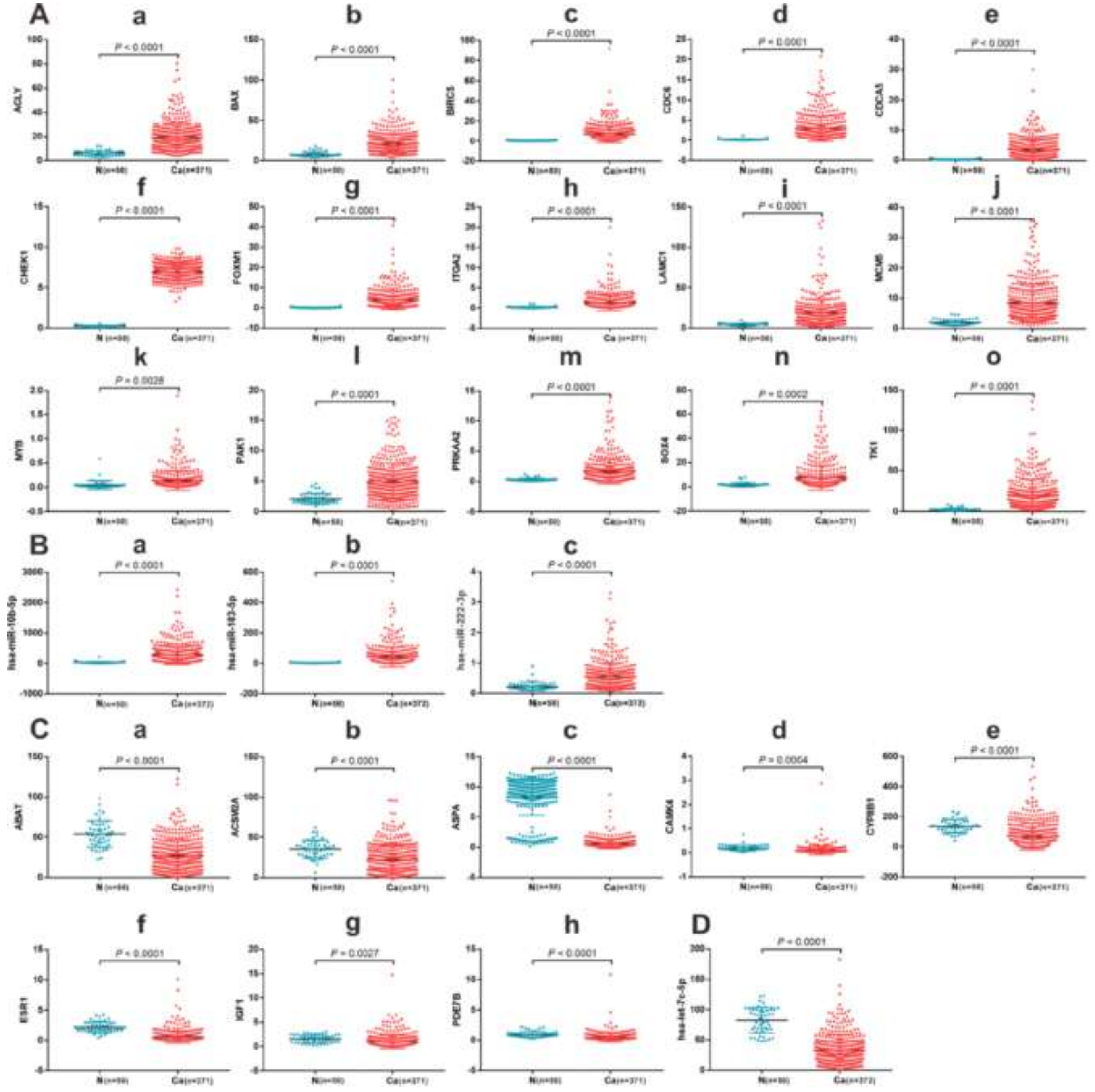


**Supplementary Figure 6. The expression level in HCC TCGA database of the DEGs and DEMs in the ceRNA network.** Upregulated DEGs in HCC tissues were ACLY, BAX, BIRC5, CDC6, CDCA5, CHEK1, FOXM1, ITGA2, LAMC1, MCM5, MYB, PAK1, PRKAA2, SOX4, TK1 (**Aa**–**Ao**). Upregulated DEMs in HCC tissues were hsa-miR-10b-5p, hsa-miR-183-5p, and hsa-miR-222-3p (**Ba**–**Bc**). Downregulated DEGs in HCC tissues were ABAT, ACSM2A, ASPA, CAMK4, CYP8B1, ESR1, IGF1, and PDE7B (**Ca**–**Ch**). Downregulated DEMs in HCC tissues were hsa-let-7c-5p (**D**).
