# Supplementary material for: Large-scale analyses identify a cluster of novel long noncoding RNAs as potential competitive endogenous RNAs in progression of hepatocellular carcinoma
Source: Aging (Albany NY). 2019 Nov 23;11(22):10422–53. doi: 10.18632/aging.102468 (PMC6914412; doi:10.18632/aging.102468)
Supplement: Supplementary Tables [file aging-11-102468-s001..pdf]

SUPPLEMENTARY TABLES

Supplementary Table 1. The top 10 upregulated and downregulated DELs in different expression profiles.

Profile	lncRNA	style	Fold Change	*P*-value	FDR
**GSE29721**	NONHSAT207339.1	up	13.563082	0.000629	0.158919
	NR_027687.1	up	10.552491	0.000232	0.097955
	NONHSAT142613.2	up	7.639081	0.00386	0.314951
	ENST00000389897.3	up	7.171353	0.000096	0.060247
	NONHSAT035498.2	up	6.544641	0.012906	0.433058
	LINC00348	up	6.469905	0.006573	0.385467
	NONHSAT165875.1	up	6.469905	0.006573	0.385467
	LINC01186	up	6.409546	0.00174	0.216378
	NONHSAT223306.1	up	6.409546	0.00174	0.216378
	SLC16A1-AS1	up	6.250394	0.001614	0.216378
	XR_430125.2	down	-30.4032	0.00042	0.062794
	LINC00844	down	-28.8462	0.002705	0.062794
	NONHSAT013506.2	down	-26.6209	0.002705	0.062794
	LINC01093	down	-26.0083	0.000273	0.062794
	NONHSAT099579.2	down	-22.2482	0.000273	0.062794
	A2MP1	down	-9.702393	0.000356	0.062794
	NONHSAT161826.1	down	-9.702393	0.000356	0.062794
	LOC105373764	down	-9.685478	0.00005	0.062794
	NONHSAT183166.1	down	-9.685478	0.00005	0.062794
	C3P1	down	-9.527459	0.001042	0.062794
**GSE40367**	NONHSAT150376.1	up	10.09334	0.002556	0.103234
	NONHSAT073632.2	up	9.626051	0.000564	0.05498
	ENST00000624682.1	up	8.135566	0.000031	0.003617
	ENST00000602609.1	up	6.688906	0.000346	0.02906
	NONHSAT093645.2	up	6.679915	0.009827	0.306808
	ENST00000602609.1	up	6.688906	0.000346	0.02906
	NONHSAT093645.2	up	6.679915	0.009827	0.306808
	OVOS2	up	6.635638	0.000953	0.075262
	NMRAL2P	up	6.128219	0.001682	0.103234
	NR_033752.2	up	6.128219	0.001682	0.103234
	NONHSAT024268.2	down	-15.590468	0.00014	0.004823
	NONHSAT121724.2	down	-13.397352	0.000076	0.004823
	XR_926922.1	down	-9.912165	0.000151	0.004823
	MAGI2-AS3	down	-9.341953333	0.000204667	0.007230667
	ENST00000562300.5	down	-9.189726	0.000515	0.014854
	LOC105370832	down	-9.189726	0.000515	0.014854
	LOC157273	down	-9.082558	0.000457	0.014854
	NONHSAT215396.1	down	-9.082558	0.000457	0.014854
	ENST00000417354.2	down	-8.714519	0.000317	0.011311
	FAM3D-AS1	down	-8.685381	0.000166	0.004823
**GSE62232**	NONHSAT214730.1	up	2.000594	0.000705	0.001847
	ENST00000588226.5	up	2.01129	0.000159	0.000548
	PARD6G-AS1	up	2.01129	0.000159	0.000548

NR_001578.1	up	2.014546	0.002505	0.005106
TDH	up	2.014546	0.002505	0.005106
NONHSAT177356.1	up	2.016526	0.00104	0.002528
LOC105371453	up	2.018036	0.000004	0.000026
XR_917739.1	up	2.018036	0.000004	0.000026
NONHSAT066299.2	up	2.03482	0.010096	0.015225
LOC105375172	up	2.035126	0.032603	0.036968
NONHSAT101639.2	down	-2.000372	0.000026	0.000122
PART1	down	-2.000372	0.000026	0.000122
LINC-PINT	down	-2.001105	0.039153	0.042336
NONHSAT213991.1	down	-2.001105	0.039153	0.042336
NR_110825.1	down	-2.001473	0.002129	0.004477
NONHSAT092521.2	down	-2.0025	0.000014	0.000072
LOC439933	down	-2.003334	0.038805	0.042034
NONHSAT150653.1	down	-2.003633	0.000053	0.000222
ENST00000578662.1	down	-2.00697	0.041058	0.043781
LOC101927539	down	-2.00697	0.041058	0.043781

Supplementary Table 2. The top 10 upregulated and downregulated DEMs in different expression profiles.

Profile	miRNA	style	Fold Change	*P*-value	FDR
**GSE36915**	hsa-miR-551b-3p	up	2.732694	0.000198	0.000553
	hsa-miR-96-5p	up	2.589663	0.000087	0.000269
	hsa-miR-10b-5p	up	2.469124	0.015412	0.014288
	hsa-miR-224-5p	up	2.435419	0.010374	0.010986
	hsa-miR-182-5p	up	2.327596	0.005243	0.006487
	hsa-miR-452-3p	up	2.301487	0.000233	0.000636
	hsa-miR-135a-5p	up	2.250943	0.009832	0.010825
	hsa-miR-515-5p	up	2.211741	0.001409	0.002642
	hsa-miR-520h	up	2.163997	0.002988	0.004427
	hsa-miR-501-5p	up	1.98213	0.003605	0.00509
	hsa-miR-203a-3p	down	-3.338125	0.000001	0.000005
	hsa-miR-375	down	-3.151798	0.00187	0.003151
	hsa-miR-1-3p	down	-2.831155	0.000031	0.000118
	hsa-miR-483-3p	down	-2.735831	0.000022	0.000092
	hsa-miR-144-3p	down	-2.731645	0.000047	0.00016
	hsa-miR-10a-5p	down	-2.715267	0.000311	0.00079
	hsa-miR-429	down	-2.536476	0.002207	0.003531
	hsa-miR-542-3p	down	-2.536104	0.000013	0.000062
	hsa-miR-30a-3p	down	-2.524737	0.000117	0.00035
	hsa-miR-200a-3p	down	-2.484562	0.000003	0.00002
**GSE74618**	hsa-miR-21-5p	up	4.65618	0.000011	0.000181
	hsa-miR-34a-5p	up	4.629343	0.000376	0.003044
	hsa-miR-155-5p	up	3.04827	0.00063	0.004483
	hsa-miR-155-5p	up	3.04827	0.00063	0.004483
	hsa-miR-532-5p	up	2.823342	0.000956	0.005882
	hsa-miR-4322	up	2.560282	0.00006	0.00075
	hsa-miR-502-3p	up	2.26894	0.000063	0.000762
	hsa-miR-1202	up	2.185917	0.001181	0.007133
	hsa-miR-1307-3p	up	2.185861	0.000303	0.002566
	hsa-miR-501-3p	up	2.140183	0.00004	0.000546
	hsa-miR-375	down	-4.040495	0.001517	0.008667
	hsa-miR-486-5p	down	-3.431948	0.000095	0.001033
	hsa-miR-378a-5p	down	-3.396752	0.000018	0.000272
	hsa-miR-497-5p	down	-2.592723	0.000066	0.000779
	hsa-miR-125b-2-3p	down	-2.469376	0.000417	0.003297
	hsa-miR-30a-3p	down	-2.173553	0.00001	0.00017
	hsa-miR-505-3p	down	-2.106977	0.000289	0.002497
	hsa-miR-122-3p	down	-2.074589	0.012504	0.043968
	hsa-miR-424-3p	down	-1.988536	0.001084	0.006604
	hsa-miR-192-3p	down	-1.956065	0.000069	0.000793

Supplementary Table 3. miRNAs and targeted intersected mRNAs in HCC.

miRNA	Gene symbol
**hsa-let-7c-5p**	ACOX1, AGXT2, ALDH6A1, C7,CYP8B1, DCN, DMD, EPHA7, ESR2, ETNK2, FXN, IDS，IGF1, LIMK2, MASP1, PPP2R2A ,SGCD, SLC10A1, TK1, VSIG4
**hsa-miR-10a-5p**	ACOX1, ADCY1, ALDH6A1, CAMK2B, ITGA2, MAT1A, PRKAA2, SGCD, TACR1
**hsa-miR-10b-5p**	ABCG2, BMPR1B, HGF, PDE11A, PDGFRA, PRKAA2, SGCD
**hsa-miR-130a-3p**	CAMK4，ESR1，FXN，GNAO1，LAMC1，PDGFRA，TRIM71
**hsa-miR-182-5p**	ACADSB，CAMK4，COL4A1，CYP1A2，DCN，ESR1，GNAO1，PDE11A，SLC4A4
**hsa-miR-183-5p**	ABAT，ABCC9，ACSM2A，AR，ARRB1，CAMK4，CYP2B6，DBT，FXN，GNAO1，IGF1，SLC4A4，STMN1
**hsa-miR-195-5p**	AR，BIRC5，CHEK1，GLS2，MASP1，MME，MYB，SEMA6D，SLC4A4
**hsa-miR-199a-5p**	AADAT，ABCG2，ADH4，AR，CBS，CHEK1，EPHA7，HOGA1，PCK1，PDE11A，PTGIS，SLC8A1
**hsa-miR-203a-3p**	FHL1，IDS，LAMC1，PLA2R1，PLCB1，PPP1R3B，SGCD
**hsa-miR-214-3p**	ACLY，ACOX1，ADCY1，APOA5，AR，BAAT，BAX，BCKDHB，CAMK2B，CDCA5，CYP2C19，CYP8B1，FOXM1，GNAO1，GPT2，IGF1，KMO，LMNA，MASP1，MASP2，NGFR，PDE4A，PRKAA2，SEMA6D，SGCD，SOCS2，TACR1
**hsa-miR-216a-5p**	ACADSB，ADCY1，BCAT1，CYP8B1，DBT，ESR2，KCNE1，LEPR，PTGIS，SGCD
**hsa-miR-222-3p**	ACOX1，CAMK4，CD4，COL4A1，DBT，FXN，IGF1，KCNE1，PDE7B，PLA2G16，PRKAA2，SEMA6D，TACR1，TRIM71
**hsa-miR-224-5p**	AR，ARRB1，AVPR1A，DBT，ESR1，GLS2，PLCB1，PRKAA2，PTGIS
**hsa-miR-30a-3p**	ADCY1，AR，ARRB1，CAMK4，CD34，ESR1，HPGD，KCNE1，PDE11A，PRKAA2，SGCD，SLC1A1，ST6GAL2，TRIM71，USP25
**hsa-miR-375**	ADCY10，ADH1B，ADH4，AR，CYP2A6，LEPR，NCAM1，PDE2A，PLCB1，PRLR，SLC8A1，SMAD6，SPAM1，TACR1，TK1
**hsa-miR-452-5p**	ACACB，ASPA，CAMK4，IDS，IL20RA，NPY1R，PPP1R3B，PRKAA2，SEMA6D，SGCD，SLC4A4，SLC8A1
**hsa-miR-455-5p**	BAAT，CAMK4，HMGCL，HOGA1，KMO，MASP1，PAK1，VSIG4
**hsa-miR-486-5p**	ASS1，INMT，KMO，LPCAT1，SOX4
**hsa-miR-497-5p**	ADCY1，ALDH6A1，BIRC5，CAMK4，CDC6，ESR2，ITGA2，KCNK5，LAMC1，MASP1，MCM5，MME，MYB，SGCD，TRIM71
**hsa-miR-505-3p**	ABAT，ACOX1，ACVR1C，ADCY1
**hsa-miR-99a-5p**	ACAA2，ADCY1，AR，CYP1A1，DAPK2，DBT，ESR2，FHL1，GHR，KCNMA1，LEPR，MASP1，PDE11A，PTGS2，SLC16A10，SLC4A4，TF，TNXB，TRIM71

Supplementary Table 4. miRNAs and targeted intersected lncRNAs in HCC.

lncRNA	miRNA
**MCM3AP-AS1**	hsa-miR-455-5p，hsa-miR-214-3p，hsa-miR-497-5p，hsa-miR-497-5p，hsa-miR-199a-5p，hsa-miR-30a-3p，hsa-miR-182-5p，hsa-miR-195-5p
**GBAP1**	hsa-miR-486-5p，hsa-miR-497-5p，hsa-miR-199a-5p，hsa-miR-182-5p，hsa-miR-224-5p，hsa-miR-195-5p，hsa-miR-30a-3p
**SLC16A1-AS1**	hsa-let-7c-5p，hsa-miR-497-5p，hsa-miR-183-5p，hsa-miR-130a-3p，hsa-miR-222-3p，hsa-miR-455-5p，hsa-miR-486-5p，hsa-miR-505-3p
**NONHSAT172507.1**	hsa-miR-10a-5p，hsa-miR-214-3p，hsa-miR-10b-5p，hsa-miR-505-3p，hsa-miR-199a-5p，hsa-miR-182-5p，hsa-miR-195-5p，hsa-miR-222-3p，hsa-miR-10a-5p，hsa-miR-455-5p
**NONHSAT191112.1**	hsa-miR-455-5p，hsa-miR-214-3p，hsa-miR-497-5p，hsa-miR-30a-3p，hsa-miR-182-5p，hsa-miR-195-5p
**PCBP1-AS1**	hsa-miR-455-5p，hsa-let-7c-5p，hsa-miR-497-5p，hsa-miR-199a-5p，hsa-miR-183-5p，hsa-miR-224-5p，hsa-let-7c-5p，hsa-miR-497-5p，hsa-miR-214-3p，hsa-miR-216a-5p，hsa-miR-203a-3p，hsa-miR-497-5p，hsa-miR-30a-3p，hsa-miR-10b-5p，hsa-miR-182-5p，hsa-miR-224-5p，hsa-miR-505-3p，hsa-miR-195-5p，hsa-miR-10a-5p，hsa-miR-216a-5p，hsa-miR-30a-3p
**LINC01128**	hsa-miR-214-3p，hsa-miR-224-5p，hsa-miR-183-5p，hsa-miR-99a-5p，hsa-miR-199a-5p，hsa-miR-497-5p，hsa-miR-216a-5p，hsa-miR-10b-5p，hsa-miR-505-3p，hsa-miR-195-5p，hsa-miR-30a-3p，hsa-miR-203a-3p，hsa-miR-497-5p，hsa-miR-10a-5p
**HAND2-AS1**	hsa-miR-216a-5p，hsa-miR-222-3p，hsa-miR-183-5p，hsa-let-7c-5p，hsa-miR-452-5p，hsa-miR-455-5p
**MAGI2-AS3**	hsa-let-7c-5p，hsa-miR-10a-5p，hsa-let-7c-5p，hsa-miR-30a-3p，hsa-miR-10b-5p，hsa-let-7c-5p，hsa-miR-183-5p，hsa-miR-195-5p，hsa-miR-222-3p，hsa-miR-455-5p，hsa-miR-452-5p，hsa-miR-130a-3p，hsa-miR-203a-3p
**C3P1**	hsa-miR-216a-5p, hsa-miR-224-5p, hsa-miR-224-5p
**NONHSAT035498.2**	hsa-let-7c-5p，hsa-miR-182-5p，hsa-miR-486-5p，hsa-miR-199a-5p，hsa-miR-452-5p
**GAS5**	hsa-let-7c-5p，hsa-miR-455-5p，hsa-miR-30a-3p，hsa-miR-452-5p
**CYTOR**	hsa-let-7c-5p，hsa-miR-455-5p，hsa-miR-182-5p，hsa-miR-497-5p
**DIO3OS**	hsa-miR-455-5p, hsa-miR-214-3p, hsa-let-7c-5p, hsa-miR-214-3p, hsa-miR-216a-5p, hsa-miR-224-5p,
**HNF4A-AS1**	hsa-miR-182-5p，hsa-miR-224-5p，hsa-miR-195-5p，hsa-miR-222-3p
**NONHSAT202693.1**	hsa-miR-452-5p，hsa-miR-224-5p，hsa-let-7c-5p，hsa-miR-10a-5p，hsa-miR-10b-5p，hsa-miR-30a-3p，hsa-miR-214-3p

Supplementary Table 5. Top 20 degree of genes in PPI network.

Gene symbol	Biotype	style	Degree
**ESR1**	mRNA	down	17
**IGF1**	mRNA	down	15
**BIRC5**	mRNA	up	12
**CD34**	mRNA	up	10
**FOXM1**	mRNA	up	9
**ITGA2**	mRNA	up	9
**AR**	mRNA	down	9
**ACLY**	mRNA	up	9
**ACACB**	mRNA	down	9
**DCN**	mRNA	down	8
**CYP2B6**	mRNA	down	8
**ABCG2**	mRNA	down	7
**CDC6**	mRNA	up	7
**BAX**	mRNA	up	7
**CHEK1**	mRNA	up	6
**TK1**	mRNA	up	6
**PDGFRA**	mRNA	down	6
**MCM5**	mRNA	up	6
**DBT**	mRNA	down	5
**HGF**	mRNA	down	5
